# Label-free proteomics identifies Calreticulin and GRP75/Mortalin as peripherally accessible protein biomarkers for spinal muscular atrophy

**DOI:** 10.1186/gm498

**Published:** 2013-10-18

**Authors:** Chantal A Mutsaers, Douglas J Lamont, Gillian Hunter, Thomas M Wishart, Thomas H Gillingwater

**Affiliations:** 1 Euan MacDonald Centre for Motor Neurone Disease Research, University of Edinburgh, Edinburgh, EH8 9XD, UK; 2 Centre for Integrative Physiology, University of Edinburgh, Hugh Robson Building, George Square, Edinburgh, EH8 9XD, UK; 3 'FingerPrints’ Proteomics Facility, College of Life Sciences, University of Dundee, Dundee, DD1 5EH, UK; 4 Division of Neurobiology, The Roslin Institute and Royal (Dick) School of Veterinary Studies, University of Edinburgh, Edinburgh, EH25 9RG, UK

## Abstract

**Background:**

Spinal muscular atrophy (SMA) is a neuromuscular disease resulting from mutations in the *survival motor neuron 1* (*SMN1*) gene. Recent breakthroughs in preclinical research have highlighted several potential novel therapies for SMA, increasing the need for robust and sensitive clinical trial platforms for evaluating their effectiveness in human patient cohorts. Given that most clinical trials for SMA are likely to involve young children, there is a need for validated molecular biomarkers to assist with monitoring disease progression and establishing the effectiveness of therapies being tested. Proteomics technologies have recently been highlighted as a potentially powerful tool for such biomarker discovery.

**Methods:**

We utilized label-free proteomics to identify individual proteins in pathologically-affected skeletal muscle from SMA mice that report directly on disease status. Quantitative fluorescent western blotting was then used to assess whether protein biomarkers were robustly changed in muscle, skin and blood from another mouse model of SMA, as well as in a small cohort of human SMA patient muscle biopsies.

**Results:**

By comparing the protein composition of skeletal muscle in SMA mice at a pre-symptomatic time-point with the muscle proteome at a late-symptomatic time-point we identified increased expression of both Calreticulin and GRP75/Mortalin as robust indicators of disease progression in SMA mice. We report that these protein biomarkers were consistently modified in different mouse models of SMA, as well as across multiple skeletal muscles, and were also measurable in skin biopsies. Furthermore, Calreticulin and GRP75/Mortalin were measurable in muscle biopsy samples from human SMA patients.

**Conclusions:**

We conclude that label-free proteomics technology provides a powerful platform for biomarker identification in SMA, revealing Calreticulin and GRP75/Mortalin as peripherally accessible protein biomarkers capable of reporting on disease progression in samples of muscle and skin.

## Background

The autosomal recessive disease spinal muscular atrophy (SMA) is caused by deficient expression of full-length survival motor neuron (SMN) protein resulting from disruption to the *survival motor neuron 1* (*SMN1*) gene [1-3]. Although SMN is ubiquitously expressed, loss of this protein has dramatic effects on the neuromuscular system, including degeneration of lower motor neurons in the ventral horn of the spinal cord, disruption of nerve-muscle connectivity at the neuromuscular junction, and pathological changes in skeletal muscle [4-9]. Disease progression, as well as symptom severity, can vary significantly between individual patients with SMA, largely dependent upon the copy number of the near-identical *SMN2* gene [3,9]. A higher copy number of *SMN2* correlates with a milder phenotype. Similarly, disease-modifying genes are known to exist that can influence the severity of a patient’s condition [10].

This detailed understanding of SMA genetics has facilitated exciting breakthroughs in pre-clinical research over the past few years, with several approaches indicating significant potential benefits in animal models of the disease. For example, experiments using gene therapy approaches to restore *SMN1* expression have yielded impressive amelioration in neuromuscular dysfunction and large increases in the lifespan of mice with SMA [11-14]. Other approaches aimed at increasing the amount of SMN protein produced by the *SMN2* gene by promoter activation or reduction of alternative splicing of SMN2 exon 7 have also shown therapeutic benefit in animal models [15-17]. As a result, there is a growing desire to undertake clinical trials in human patient cohorts in order to evaluate the potential benefits of these therapeutic approaches. However, performing clinical trials in cohorts of young patients (and in the case of severe forms of SMA, neonatal patients) brings with it a range of technical problems [18].

In order to improve the reliability and effectiveness of SMA clinical trials, robust biomarkers are required. Firstly, biomarkers are needed to allow accurate monitoring of disease activity and to predict disease progression in human patients [19]. Secondly, biomarkers are required to provide more accurate measures of the responses of individual patients and groups of patients to a new treatment or therapeutic approach [20]. Several different approaches have previously been employed in an attempt to identify biomarkers for SMA in both mouse models and patient cohorts, incorporating a range of physical, functional and molecular readouts [19,21-23]. However, robust biomarkers for SMA have yet to be identified.

Proteomics technologies have recently been highlighted as a potentially powerful tool for biomarker discovery [20]. In this study, we have utilized a state-of-the-art, label-free proteomics approach to identify individual proteins in the neuromuscular system of SMA mice that report directly on disease status. By comparing the protein composition of skeletal muscle in SMA mice at a pre-symptomatic time-point with the muscle proteome at a late-symptomatic time-point we identified increased expression of both Calreticulin and GRP75/Mortalin as robust indicators of disease progression. We report that these protein biomarkers were similarly modified across two different mouse models of SMA, across multiple skeletal muscles, and also in skin biopsies. Furthermore, initial investigation of Calreticulin and GRP75/Mortalin levels in muscle biopsy samples suggested that these proteins are detectable and measurable by western blot in tissue from human SMA patients.

## Methods

### Mice

Two SMA mouse models were used (both on a congenic FVB background). The 'severe’ SMA mouse model [24] (*Smn-/-;SMN2tg/tg*) was originally obtained from the Jackson Laboratory (Bar Harbor, Maine, USA) and had a mean survival of 5/6 days in our hands. The 'Taiwanese’ SMA mouse model (*Smn-/-;SMN2tg/0*) [25] was also obtained from Jackson Laboratories and was maintained following the breeding strategy devised by Riessland and colleagues [26], giving a mean survival of 10/11 days. Litters produced from both 'severe’ SMA and 'Taiwanese’ SMA mice were retrospectively genotyped using standard PCR protocols (JAX® Mice Resources; the Jackson Laboratory (Bar Harbor, Maine, USA)), as previously described [5,26]. All animal procedures and breeding were performed in accordance with Home Office guidelines in the UK.

### Human muscle samples

*Quadriceps femoris* biopsy samples were obtained, through EuroBioBank [27], from two different biobanks in Italy; Fondazione IRCCS Istituto Neurologico 'C Besta’ in Milan and Fondazione Ospediale Maggiore Polclinico Mangiagalli en Regina Elena, IRCCS in Milan. All required ethical approvals to acquire and distribute human patient tissue samples were obtained by the host biobanks. Tissue was shipped to Edinburgh in an anonymous fashion, with no identifying details provided apart from the age, gender and genetic status of the patients. Biopsies were obtained from three type II/III SMA patients (aged between 3 and 25 years), with a homozygous deletion of the *SMN1* gene confirming a genetic diagnosis of SMA. Three age-matched control samples were also obtained, genetically confirmed to have no mutations in the *SMN1* gene.

### Mouse sample preparation

'Severe’ SMA mice (*Smn-/-;SMN2*+/+) and wild-type (*Smn+/+;SMN2*+/+) littermates at postnatal day 1 (P1) and P5 were sacrificed by chilling on ice and decapitation. *Levator auris longus* (LAL; from the back of the neck) muscles were dissected in oxygenated mammalian physiological saline, as previously described [28]. LAL muscles were separated into rostral and caudal bands and quickly frozen on dry ice. The rostral band of LAL from each mouse was stored at -80°C until sufficient tissue was collected for proteomics analysis.

'Taiwanese’ SMA mice and littermate controls were sacrificed at P1, P5, P7 and P9 before the *gastrocnemius* muscle was dissected from each hind limb. At the same time, a sample of skin from the belly was taken and a few drops of blood were collected. All tissue was quickly frozen on dry ice and stored in -80°C freezers for further analysis.

### Label-free proteomics

Protein was extracted in MEBC buffer (50 mM TRIS, 100 mM NaCl, 5 mM NaEDTA, 5 mM NaEGTA, 40 mM β-glycerophosphate, 100 mM NaF, 100 mM sodium orthovanadate, 0.25% NP-40, 1 Roche 'complete’ protease inhibitor tablet, pH 7.4). Protein concentration was determined by bicinchoninic acid assay (BCA; Thermo Scientific Pierce, Rockford, IL, USA) according to the manufacturer's instructions on solubilised muscle (P1 wild-type and SMA rostral and P5 wild-type and SMA rostral). Then 10 μg aliquots of each muscle type were reduced with 10 mM dithiothreitol and alkylated with 50 mM iodoacetamide prior to digestion with trypsin (sequencing grade; Roche, Indianapolis, IN, USA) overnight at 30°C. Technical replicates (3 × 2.5 μg) of each digested muscle type were injected onto a nano-scale liquid chromatographic tandem mass spectrometry (nLC-MS/MS) system (Ultimate 3000 (Dionex (Thermo Fisher), Hemel Hempstead, UK) coupled to a LTQ Orbitrap XL (Thermo Scientific, Hemel Hempstead, UK). The peptides from each digest were separated over a 65 minute linear gradient from 5 to 35% acetonitrile in 0.1% formic acid. The LTQ Orbitrap XL was configured with a TOP 5 methodology comprising a 60 K resolution FT-MS full scan followed by IT-MS/MS scans for the 5 most intense peptide ions. The raw data were then imported into Progenesis LCMS for label free differential analysis and subsequent identification and quantification of relative ion abundance ratios, both up-regulated and down-regulated. Following alignment of MS data, principal component analysis and preliminary filtering (power >80%, *P* > 0.05), data were exported from Progenesis as a single mgf file per time-point. These files were then used to identify individual peptide sequences using the Swiss-Prot database via Mascot Daemon (v2.4.0) due to the large file size. As an indication of identification certainty, the false discovery rate for peptide matches above identity threshold was 9.39% for P1 and 3.34% for P5. Protein abundance data per experimental run/sample as an output from the Progenesis software can be found in Additional file 1 and abundance of individual peptides can be found in Additional file 2. Statistical *P*-values presented in Tables 1 and 2 and Additional file 1 were automatically generated using Progenesis software through a one-way Anova on the ArcSinh transform of the normalised data.

**Table 1:** **Proteins unchanged at P1 but increased >50**% **or 20 to 50**% **at P5 in the rostral band of LAL muscle from SMA mice compared with littermate controls**

**Gene name**	**Protein name**	**Accession number**	**Peptides at P5**	**Ratio KO/control P1**	**Ratio KO/control at P5**	**Score at P5**	**Anova ( *P *-value) at P5**
*G6pI*	Glucose-6-phosphate isomerase	G6PI_MOUSE	6	1.03	1.98	245.68	6.92E-06
*Grp75*	Stress-70 protein	GRP75_MOUSE	3	1.02	1.82	170.83	5.59E-04
*Tbb5*	Tubulin beta-5 chain	TBB5_MOUSE	2	1.02	1.80	639.05	3.69E-05
*Plec1*	Plectin-1	PLEC1_MOUSE	21	1.18	1.63	879.72	4.67E-06
*Thil*	Acetyl-CoA acetyltransferase	THIL_MOUSE	7	1.09	1.59	448.39	2.95E-04
*Calr*	Calreticulin	CALR_MOUSE	3	1.12	1.51	83.76	2.08E-03
*Aatm*	Aspartate aminotransferase	AATM_MOUSE	7	1.17	1.50	443.54	1.50E-04
*Thim*	3-Ketoacyl-CoA thiolase	THIM_MOUSE	12	1.02	1.41	473.14	1.16E-03
*Anxa2*	Annexin A2	ANXA2_MOUSE	10	1.04	1.33	729.11	1.28E-03
*Pura1*	Adenylosuccinate synthetase isozyme 1	PURA1_MOUSE	5	1.09	1.30	247.24	1.19E-03
*S10ab*	Protein S100-A11	S10AB_MOUSE	2	1.01	1.25	166.21	2.78E-02
*Hbb2*	Hemoglobin subunit beta-2	HBB2_MOUSE	6	1.01	1.23	702.27	1.51E-03
*Lmna*	Lamin-A/C	LMNA_MOUSE	7	1.16	1.21	567.31	2.19E-04
*Vime*	Vimentin	VIME_MOUSE	12	1.17	1.21	776.2	3.50E-04

KO, knockout.

**Table 2:** **Proteins unchanged at P1 but decreased >50**% **or 20 to 50**% **at P5 in the rostral band of LAL muscle from SMA mice compared with littermate controls**

**Gene name**	**Protein name**	**Accession number**	**Peptides at P5**	**Ratio KO/control at P1**	**Ratio KO/control at P5**	**Score at P5**	**Anova ( *P *-value) at P5**
*Rrbp1*	Ribosome-binding protein 1	RRBP1_MOUSE	3	0.89	0.15	60.23	1.97E-05
*Tpm1*	Tropomyosin alpha-1 chain	TPM1_MOUSE	6	0.92	0.25	652.67	6.87E-06
*Actn2*	Alpha-actinin-2	ACTN2_MOUSE	15	0.89	0.61	1413.57	1.82E-04
*Tcpb*	T-complex protein 1 subunit beta	TCPB_MOUSE	3	0.89	0.66	162.09	2.07E-03
*Postn*	Periostin	POSTN_MOUSE	3	0.99	0.70	142.66	7.55E-04
*Hs90a*	Heat shock protein HSP 90-alpha	HS90A_MOUSE	3	0.99	0.70	326.05	7.71E-04
*Hs90b*	Heat shock protein HSP 90-beta	HS90B_MOUSE	8	0.90	0.70	600.51	6.45E-05
*Rtn4*	Reticulon-4	RTN4_MOUSE	5	0.86	0.79	123.82	1.69E-02
*Nb5r3*	NADH-cytochrome b5 reductase 3	NB5R3_MOUSE	3	0.95	0.79	65.43	3.90E-05

KO, knockout.

These data from Mascot were then re-imported into Progenesis for subsequent conflict resolution and protein expression comparison. Stringent selection criteria were used before a protein was included in our analyses; identification of at least two peptides was needed and a *P*-value <0.05 [29,30]. To be identified as a protein with changed expression levels in SMA tissue, the protein had to be up- or down-regulated by >20% compared to wild-type controls. The mass spectrometry proteomics data have been deposited in the ProteomeXchange Consortium [31] via the PRIDE partner repository with the dataset identifier PXD000488 and DOI10.6019/PXD000488.

### Quantitative fluorescent western blotting

Protein was extracted from 'Taiwanese’ SMA mouse muscle, skin and blood samples and from human muscle biopsies. Protein levels were quantified by BCA. Quantitative western blots were performed as described previously [30,32]. Briefly, the membranes were put in 2% Ponceau S for 10 minutes and then washed briefly in ddH_2_O until the bands were clearly visible and the background stain low. Then the membranes were blocked in buffer for 30 minutes before incubating in primary antibodies against Calreticulin (1:1,000; Lifespan Biosciences, Seattle, WA, USA), GRP75 (1:2,500; Lifespan Biosciences) or TCP1 beta (1:1,000; Abcam, Cambridge, UK). Odyssey secondary antibodies were added according to the manufacturer's instructions (Goat anti rabbit IRDye 680 or 800, Goat anti mouse IRDye 680 or 800 and Donkey anti Goat IRDye 800 dependent on required combinations; LI-COR Biosciences, Cambridge, UK). Blots were imaged using an Odyssey Infrared Imaging System (LI-COR Biosciences, Cambridge, UK) at 169 μm resolution. Where possible, each sample was independently run and measured twice to minimise user variability.

### Statistical analysis

All data were collected into Microsoft Excel spreadsheets and then analysed using GraphPad Prism software. For all statistical analyses *P* < 0.05 was considered to be significant. Individual statistical tests used are detailed in the results section or figure legends.

## Results

### Label-free proteomics analysis reveals a list of 23 putative biomarkers of disease status in skeletal muscle from 'severe’ SMA mice

In order to identify potential new protein biomarkers capable of reporting directly on the progress of disease in SMA, we utilized unbiased, label-free proteomics technologies to compare the proteome of a pathologically affected tissue in SMA (skeletal muscle) [7] at early- and late-symptomatic stages of the disease. Given the difficulty in obtaining human muscle samples for such an experiment, we performed these initial proteomic screens in the LAL muscle of an established mouse model of SMA (the 'severe’ SMA mouse; *Smn-/-;SMN2+/+*;) [24]. The mouse LAL muscle is composed of two distinct muscle bands that are differentially affected in SMA mice: a caudal band that undergoes severe neuromuscular denervation [5,33] and a rostral band that has minimal denervation but intrinsic muscular pathology [7]. In order to obtain a pathologically homogeneous tissue sample for proteomics analysis, we chose to selectively examine the larger rostral band of the muscle.

The rostral band of LAL muscle was dissected from 'severe’ SMA mice and littermate controls (*Smn+/+;SMN2+/+*) at P1 (pre/early-symptomatic) and P5 (n = 9 mice per genotype, per time-point) and proteins were extracted for mass-spectrometry analysis. The raw data from P1 animals was previously analysed using the IPI mouse database and was published [7]. For the current study, to allow direct comparison with the P5 data, the P1 raw data were re-analysed in parallel with the P5 data as detailed below.

The raw mass-spectrometry data from both P1 and P5 comparisons were uploaded to Progenesis label-free software for further analyses. From each sample three replicate runs were performed. One control replicate was chosen as a reference dataset, based on a clear and representative feature pattern with minimum distortion. All other runs were then aligned to this reference dataset using the Progenesis software. Alignment was performed to correct for the variable elution of peptides during chromatographic separation. Although Progenesis software automatically aligns data from each experimental run, vectors were also added manually to align peptide ions where needed. After aligning data from each of the runs, data filtering was performed. All ions that were identified with an early (less than 6 minutes) or late retention time (more than 72 minutes) were excluded. The runs were then grouped according to the genotype of the mouse (for example, into pooled control and SMA datasets) and an ANOVA statistical test was performed to determine whether the means of the two groups were equal. At this stage further stringent filtering was performed and all 1^+^ charged ions were excluded as unlikely to represent peptides.

**Figure 1:**
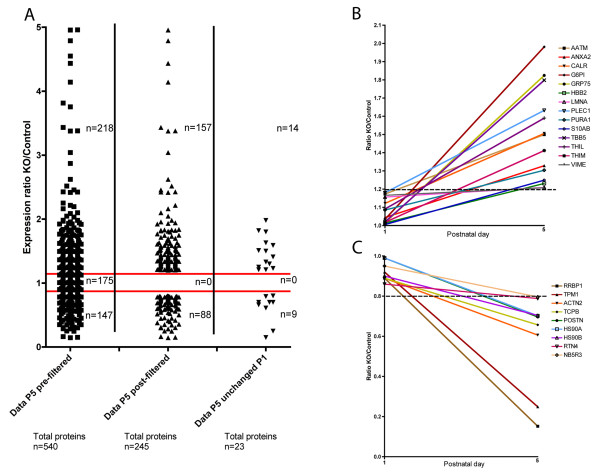
**Identification of putative protein biomarkers for SMA in skeletal muscle from 'severe’ SMA mice. (A)** Scatter plot showing the process of filtering undertaken on the raw proteomics data in order to generate a final list of 23 proteins modified in SMA mouse skeletal muscle at P5, but unchanged at P1. The left column shows all proteins identified by the Progenesis label-free proteomics software (n = 540 proteins in total) in control and SMA (knockout (KO)) mouse LAL muscle at P5, with the relative expression levels between samples represented as a ratio (KO/Control). The red bars indicate the 20% cutoff threshold for being up-regulated or down-regulated in SMA mice compared to controls. The middle column shows the proteins remaining in the P5 dataset following filtering (for example, that were either up- or down-regulated by >20% and were identified by at least two peptides (n = 245 proteins in total)). The right column shows those proteins that were identified as being changed in SMA mouse skeletal muscle at P5, but that were unchanged in comparable muscle samples at P1 (n = 23 proteins in total). **(B)** Graph showing all 14 proteins that were unchanged at P1 in 'severe’ SMA mouse LAL muscle compared to littermate controls, but had increased levels >20% at P5. **(C)** Graph showing all nine proteins that were unchanged at P1 in 'severe’ SMA mouse LAL muscle compared to littermate controls, but had decreased levels >20% at P5. Dashed lines in B and C indicate 20% change cut-off thresholds.

Once the list of candidate peptide ions to identify was created, their MS/MS data were exported to the Swiss-Prot protein database to allow comparison to known peptides, and subsequently proteins. The output of this Swiss-Prot analysis was then re-imported to Progenesis software in order to allow further filtering by excluding peptides that were not associated with more than one protein (conflict resolving). Also, peptides that were improperly cleaved by trypsin were excluded (for example, any peptide that had a lysine or arginine mid-sequence, or any peptides not ending with a lysine or arginine). This led to the identification of 540 proteins in the P5 dataset (Figure 1A, left column). A filtering protocol was then applied for subsequent stringent positive identification of proteins, with only those proteins identified by two or more unique peptides taken forward for further analysis. Proteins that were either up- or down-regulated >20% in SMA muscle compared to controls were considered to have an altered expression profile (Figure 1A, middle column).

To be considered as a putative biomarker, we wanted to identify proteins whose expression levels were unchanged in SMA muscle at P1 (pre/early-symptomatic), but were significantly changed at P5 (late-symptomatic). We therefore took the list of all proteins with modified expression in SMA mice at P5 compared to controls, and searched for expression data for the same proteins in the P1 comparison dataset. Any proteins found to have altered expression at both P5 and P1 were considered to be unsuitable as a biomarker and were therefore removed from the candidate list (Figure 1A, right column). This filtering of data resulted in the identification of 14 candidate biomarker proteins that were up-regulated in 'severe’ SMA mouse muscle at P5 but not at P1 (Table 1, Figure 1B) and 9 proteins that were down-regulated in 'severe’ SMA mouse muscle at P5 but not at P1 (Table 2, Figure 1C).

### Validation of putative protein biomarkers in the 'Taiwanese’ mouse model of SMA

In order to validate the list of candidate biomarkers generated by our proteomics analysis of skeletal muscle from 'severe’ SMA mice, we wanted to determine whether similar changes in protein levels could be detected in a different skeletal muscle from a genetically unique mouse model of SMA using quantitative fluorescent western blotting. We chose three proteins to validate, based on the magnitude of their expression change and the availability of suitable antibodies for western blotting: Stress-protein 70 (GRP75/Mortalin) and Calreticulin were 1.8- and 1.5-fold up-regulated, respectively, in our proteomic dataset whereas T-complex protein 1 subunit beta (TCP1) was down-regulated by 1.5-fold. We measured levels of these three proteins in the *gastrocnemius* muscle (from the hind limb) of 'Taiwanese’ SMA mice and littermate controls [7,26].

**Figure 2:**
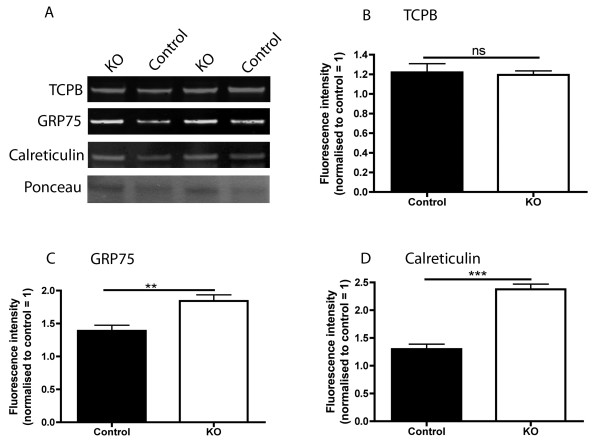
**Validation of Calreticulin and GRP75/Mortalin as potential protein biomarkers in a different muscle from a genetically distinct SMA mouse model. (A)** Representative fluorescent western blots on gastrocnemius muscle from 'Taiwanese’ SMA mice (knockout (KO)) and littermate controls at P9 (mid/late-symptomatic) showing levels of TCPB, GRP75/Mortalin, Calreticulin and Ponceau (loading control). **(B-D)** Bar charts (mean ± standard error of the mean) showing expression levels of TCPB, GRP75/Mortalin and Calreticulin in control and 'Taiwanese’ SMA mice (KO) at P9 (N = 3 mice per genotype). TCPB levels showed no difference in expression levels in SMA mice compared with controls (ns, not significant; *P* > 0.05, unpaired, two-tailed *t*-test) **(B)**. GRP75 levels were significantly increased in 'Taiwanese’ SMA mice compared with controls (**P < 0.01, unpaired, two-tailed *t*-test) **(C)**. Calreticulin levels were also significantly increased in Taiwanese-SMA mice compared with controls (***P < 0.001, unpaired, two-tailed *t*-test) **(D)**.

Levels of TCP1, GRP75/Mortalin and Calreticulin were measured in the *gastrocnemius* muscle of 'Taiwanese’ SMA mice and littermate controls at a mid/late-symptomatic time-point (P9; Figure 2A). Levels of TCP1 were unchanged in the SMA mice compared to controls (Figure 2B), thereby failing to validate the original proteomics data in a different model of SMA. However, in contrast, levels of both GRP75/Mortalin and Calreticulin were significantly increased in the 'Taiwanese’ SMA mouse muscle, showing that the changes in these proteins were conserved between 'severe’ and 'Taiwanese’ SMA mice, as well as between the LAL and *gastrocnemius* muscles (Figure 2B,C).

### Preliminary investigation of a small patient cohort suggests that levels of GRP75/Mortalin and Calreticulin are increased in human SMA patient muscle biopsies

Next, we wanted to establish whether the increased levels of GRP75/Mortalin and Calreticulin, observed to correlate with disease progression in SMA mouse models, were also measurable in skeletal muscle from human SMA patients. Therefore, we examined levels of GRP75/Mortalin and Calreticulin using quantitative fluorescent western blotting on human muscle biopsy samples obtained through EuroBioBank (see Methods). We obtained biopsies from the *quadratus femoris* from three type II/III SMA patients (aged between 3 and 25 years). All three patients had a genetic diagnosis of SMA confirmed by a homozygous deletion of the *SMN1* gene. Three roughly age-matched control samples were also obtained, genetically confirmed to have no mutations in the *SMN1* gene.

**Figure 3:**
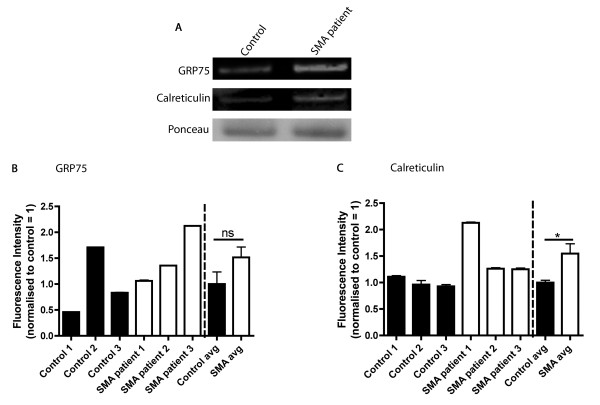
**GRP75/Mortalin and Calreticulin are measurable in muscle biopsies from human SMA patients. (A)** Representative fluorescent western blots on *quadriceps femoris* muscle biopsy samples from an SMA patient (type II/III) and an age-matched non-SMA control showing levels of GRP75/Mortalin, Calreticulin and Ponceau (loading control). **(B,C)** Bar charts showing expression levels of GRP75 and Calreticulin in human SMA patient muscle biopsies compared with controls. Data are shown for each individual patient (black and white bars to the left of the dashed line; error bars show variability between two independent measurements taken from that individual’s biopsy), as well as pooled mean for each patient group (right of the line; ± standard error of the mean; n = 6 measurements for each group, two independent measurements from each patient biopsy). **(B)** GRP75 levels showed a trend towards increased expression in SMA patients, but this difference did not reach statistical significance (ns, not significant; *P* > 0.05, unpaired, two-tailed *t*-test). **(C)** Calreticulin levels were significantly increased in SMA patient muscle (*P < 0.05, unpaired, two-tailed *t*-test).

Both GRP75/Mortalin and Calreticulin could be readily identified and levels measured using quantitative fluorescent western blotting. Both GRP75/Mortalin and Calreticulin showed a trend towards increased levels in the small cohort of SMA patients compared to controls (Figure 3). GRP75/Mortalin levels increased on average by 50% compared to controls, although the considerable variability between individuals and low sample size meant that this difference did not reach statistical significance (Figure 3B). Calreticulin levels were significantly increased in the SMA patient biopsies, on average by 50% compared to controls (Figure 3C); however, there was still considerable variability between individuals. Whilst these experiments only represent an initial attempt to measure GRP75/Mortalin and Calreticulin levels in human patient muscle biopsies, and are limited by the very small sample size, our preliminary investigations suggest that both Calreticulin and GRP75/Mortalin may represent accessible protein biomarkers in skeletal muscle conserved between mouse models and human patients.

### Altered levels of GRP75/Mortalin and Calreticulin can be detected in skin biopsies from SMA mice

**Figure 4:**
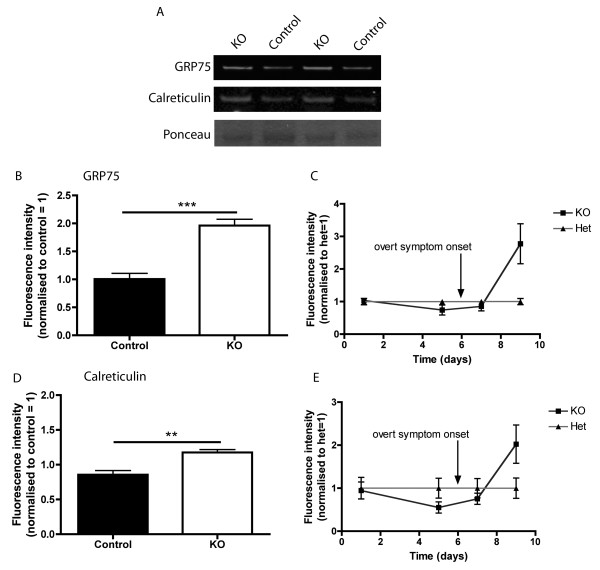
**Increased levels of Calreticulin and GRP75/Mortalin in skin biopsies correlate with disease progression in SMA mice. (A)** Representative fluorescent western blots on skin tissue from 'Taiwanese’ SMA mice and littermate controls at P9 (mid/late-symptomatic) showing levels of GRP75/Mortalin, Calreticulin and Ponceau (loading control). **(B,D)** Bar charts (mean ± standard error of the mean) showing expression levels of GRP75/mortalin and Calreticulin in 'Taiwanese’ SMA mice (knockout (KO)) and controls at P9 (N = 3 mice per genotype). **(B)** GRP75/Mortalin levels were significantly increased in 'Taiwanese’ SMA mice compared with controls (***P < 0.01, unpaired, two-tailed *t*-test). **(D)** Calreticulin levels were significantly increased in Taiwanese SMA mice compared with controls (**P < 0.01, unpaired, two-tailed *t*-test). **(C,E)** Time course of GRP75/Mortalin and Calreticulin expression in skin biopsies from 'Taiwanese’ SMA mice (KO) compared to controls (Het) (N = 3 mice per genotype/time-point). Tissue was analysed in mice at P1, P5 (both pre/early-symptomatic), P7 (early-symptomatic) and P9 (mid/late-symptomatic). **(C)** There was no increase in GRP75/Mortalin levels in Taiwanese SMA mice until after overt disease onset. **(E)** Similarly, there was no increase in Calreticulin levels in Taiwanese SMA mice until after overt disease onset.

Our analyses of GRP75/Mortalin and Calreticulin levels in skeletal muscle from SMA mouse models (supported by preliminary investigations of human patient tissue) suggested that these two proteins may represent robust protein biomarkers for SMA. However, obtaining muscle biopsies from human patients is an invasive procedure that is not ideal for repeated analyses of protein levels during a clinical trial, especially in small children. As a result, the availability of biomarker proteins in more peripherally accessible tissue (such as skin and/or blood) would make it much easier to obtain quick, repeated tissue samples for monitoring purposes. Therefore, we next asked whether GRP75/Mortalin and Calreticulin protein could be reliably identified and measured in skin and blood. Analysis of expression datasets [34] confirmed that both GRP75/Mortalin and Calreticulin are known to be expressed in skin and whole blood. To establish whether these proteins were detectable in skin and blood samples from our mouse models we performed standard quantitative fluorescent western blotting for both of these proteins on samples taken from 'Taiwanese’ SMA mice and littermate controls at P9. Neither GRP75/Mortalin nor Calreticulin could be reliably detected in whole blood (data not shown). However, both proteins were robustly expressed in skin samples, with their levels being significantly increased in SMA mice compared to controls (Figure 4). Thus, both GRP75/Mortalin and Calreticulin were readily identifiable in skin biopsies, with the alterations in their levels in skin closely matching alterations previously observed in skeletal muscle (Figure 4B,D).

Finally, we wanted to establish whether GRP75/Mortalin and Calreticulin levels in skin matched the temporal profile we originally identified in our muscle proteomics experiments. Therefore, we collected skin samples from 'Taiwanese’ SMA mice and littermate controls at four different time-points: P1 and P5 (pre-symptomatic), P7 (early-symptomatic), and P9 (mid/late-symptomatic). Temporal changes in the levels of GRP75/Mortalin and Calreticulin showed similar trends in SMA mouse skin, with no differences observed at pre/early-symptomatic time-points, but robust increases evident after onset of symptoms at P6 (Figure 4C,E). Thus, the temporal expression of GRP75/Mortalin and Calreticulin revealed a very similar profile in skin to that previously observed in skeletal muscle. Once again, the robust increases in expression correlated with disease progression, confirming that GRP75/Mortalin and Calreticulin represent peripherally accessible protein biomarkers capable of reporting on disease status in SMA.

## Discussion

In this study we used label-free proteomic technology to identify two proteins with the potential to act as molecular biomarkers for SMA. The combination of proteomics technology with an established mouse model of SMA (where it is possible to accurately identify and isolate tissue from animals at different stages of disease) revealed that increased levels of GRP75/Mortalin and Calreticulin in skeletal muscle correlated with disease progression. Importantly, these protein biomarkers were also accessible in skin samples from SMA mice, suggesting that they can also be monitored in a peripherally accessible tissue during clinical trials. A preliminary study on a small sample of patient muscle biopsies suggested that GRP75/Mortalin and Calreticulin were detectable and measurable in human tissue, including biopsies from SMA patients.

The use of label-free proteomics in this study provides further evidence that proteomics technologies represent a powerful tool for biomarker discovery [20]. Indeed, proteomics technology has previously been used to screen for potential biomarkers in human SMA patients [19,21]. Previous studies combining proteomics with animal models of SMA have mainly utilized the technology to uncover molecular pathways disrupted downstream of SMN [7,30,35,36], but the current study demonstrates that similar approaches can be used to identify potential protein biomarkers for future use in the human clinical context. In addition, our ability to identify protein biomarkers conserved between different mouse models of SMA and human SMA patients suggests that common biomarkers can be utilized in both pre-clinical testing of new treatments in animal models as well as in human clinical trials. It should be noted that our proteomics study identified approximately 500 muscle proteins, which is predicted to represent only a fraction of the total muscle proteome. Thus, there are likely to be other proteins in SMA skeletal muscle yet to be identified that have the potential to act as novel biomarkers for the disease alongside GRP75/Mortalin and Calreticulin.

Calreticulin is a multifunctional protein that has previously been identified as a potential biomarker for other diseases. For example, serum levels of Calreticulin have been shown to increase in patients with rheumatoid arthritis [37] and increased levels of Calreticulin have been reported in breast cancer [38,39], gastric cancer [40], and lung cancer [41]. Calreticulin has also been identified as a prognostic factor for neuroblastoma [42]. However, Calreticulin has not previously been linked to SMA, and whether it is actively involved in disease pathogenesis or not remains unclear. Interestingly, Calreticulin has been implicated in regulating motor neuron pathology in a related motor neuron disease (amyotrophic lateral sclerosis; ALS) [43], suggesting that, alongside its potential to act as a molecular biomarker for SMA, further investigations into its possible contribution to the pathogenesis of SMA are warranted.

GRP75/Mortalin is a member of the Hsp70 family of chaperones, with roles including the regulation of energy generation, stress responses, muscle activity, mitochondrial activity, and cellular viability [44-46]. As with Calreticulin, GRP75/Mortalin has previously been identified as a possible biomarker for cancer and cardiovascular diseases [47] as well as being a potential prognostic factor for neuroblastoma [48]. GRP75/Mortalin (also known as HSPA9) has also been implicated in the pathogenesis of other neurodegenerative conditions, including Parkinson’s disease [49] and Alzheimer’s disease [50], suggesting that it, too, may be contributing directly to SMA pathogenesis. Nevertheless, it is important to note that biomarkers do not need to actively contribute to disease pathogenesis in order to be effective. What is critical is that the levels of a biomarker must alter in a temporally traceable and predictable manner as an accurate measure of the molecular and physiological processes of disease progression. Both GRP75/Mortalin and Calreticulin appear to meet these criteria in SMA.

Our preliminary investigation of GRP75/Mortalin and Calreticulin levels in human skeletal muscle suggests that these proteins may represent viable biomarkers in human SMA patients. However, it represents only an initial demonstration of the ability to detect and measure these proteins in human tissue, and was hampered by a lack of detailed information from the biobank regarding the actual stage of disease progression for each patient at the time of muscle biopsy, as well as the small sample size. As a result, further, large-scale studies on patient cohorts will now be required to validate GRP75/Mortalin and Calreticulin as robust protein biomarkers for SMA in humans. The demonstration in mouse models that increased levels of these proteins correlated with increasing disease severity suggests that such a study is now warranted. Moreover, the finding that these proteins can be tracked in skin samples suggests that the use of skin biopsies might be of more practical use for these studies, reducing the need for repeated invasive muscle biopsies.

## Conclusions

We conclude that label-free proteomics technology provides a powerful platform for biomarker identification in SMA. Combining label-free proteomics with established mouse models of SMA led to the identification of Calreticulin and GRP75/Mortalin as protein biomarkers capable of reporting on disease progression in tissue samples of muscle and skin. When used alongside the genetic *SMN* status of SMA patients, these biomarkers should provide an additional means through which the disease can be monitored and tracked. Further work is now warranted to validate these protein biomarkers in cohorts of SMA patients.

## Abbreviations

LAL: *Levator auris longus*; LC: Liquid chromatography; MS/MS: Tandem mass spectrometry; P: Postnatal day; SMA: Spinal muscular atrophy; SMN: Survival motor neuron.

## Competing interests

The authors declare that they have no competing interests.

## Authors’ contributions

CAM, TMW and THG designed the study; CAM DJL, GH, TMW and THG performed experiments and analysed data; CAM, TMW and THG wrote the manuscript; all authors read and approved the final manuscript.

## Supplementary Material

Additional file 1: Table S1 Protein abundance output from Progenesis analysis software for all identified proteins from P5 SMA versus control animals. Click here for file

Additional file 2: Table S2 Peptide abundance (feature) output from Progenesis analysis software for proteins from P5 SMA versus control animals. Click here for file

## References

[B1] Lefebvre S Burglen L Reboullet S Clermont O Burlet P Viollet L Benichou B Cruaud C Millasseau P Zeviani M Identification and characterization of a spinal muscular atrophy-determining gene Cell 1995 5 155 165 10.1016/0092-8674(95)90460-3 7813012

[B2] Burghes AH Beattie CE Spinal muscular atrophy: why do low levels of survival motor neuron protein make motor neurons sick? Nat Rev Neurosci 2009 5 597 609 19584893 10.1038/nrn2670 PMC2853768

[B3] Lorson CL Rindt H Shababi M Spinal muscular atrophy: mechanisms and therapeutic strategies Hum Mol Genet 2010 5 R111 R118 10.1093/hmg/ddq147 20392710 PMC2875050

[B4] Hamilton G Gillingwater TH Spinal muscular atrophy: going beyond the motor neuron Trends Mol Med 2013 5 40 50 10.1016/j.molmed.2012.11.002 23228902

[B5] Murray LM Comley LH Thomson D Parkinson N Talbot K Gillingwater TH Selective vulnerability of motor neurons and dissociation of pre- and post-synaptic pathology at the neuromuscular junction in mouse models of spinal muscular atrophy Hum Mol Genet 2008 5 949 962 18065780 10.1093/hmg/ddm367

[B6] Lunn MR Wang CH Spinal muscular atrophy Lancet 2008 5 2120 2133 10.1016/S0140-6736(08)60921-6 18572081

[B7] Mutsaers CA Wishart TM Lamont DJ Riessland M Schreml J Comley LH Murray LM Parson SH Lochmuller H Wirth B Talbot K Gillingwater TH Reversible molecular pathology of skeletal muscle in spinal muscular atrophy Hum Mol Genet 2011 5 4334 4344 10.1093/hmg/ddr360 21840928

[B8] Ling KK Gibbs RM Feng Z Ko CP Severe neuromuscular denervation of clinically relevant muscles in a mouse model of spinal muscular atrophy Hum Mol Genet 2012 5 185 195 10.1093/hmg/ddr453 21968514 PMC3235013

[B9] Swoboda KJ Prior TW Scott CB McNaught TP Wride MC Reyna SP Bromberg MB Natural history of denervation in SMA: relation to age, SMN2 copy number, and function Ann Neurol 2005 5 704 712 10.1002/ana.20473 15852397 PMC4334582

[B10] Oprea GE Krober S McWhorter ML Rossoll W Muller S Krawczak M Bassell GJ Beattie CE Wirth B Plastin 3 is a protective modifier of autosomal recessive spinal muscular atrophy Science 2008 5 524 527 10.1126/science.1155085 18440926 PMC4908855

[B11] Foust KD Wang X McGovern VL Braun L Bevan AK Haidet AM Le TT Morales PR Rich MM Burghes AH Kaspar BK Rescue of the spinal muscular atrophy phenotype in a mouse model by early postnatal delivery of SMN Nat Biotechnol 2010 5 271 274 10.1038/nbt.1610 20190738 PMC2889698

[B12] Valori CF Ning K Wyles M Mead RJ Grierson AJ Shaw PJ Azzouz M Systemic delivery of scAAV9 expressing SMN prolongs survival in a model of spinal muscular atrophy Sci Transl Med 2010 5 35ra42 10.1126/scitranslmed.3000830 20538619

[B13] Bevan AK Hutchinson KR Foust KD Braun L McGovern VL Schmelzer L Ward JG Petruska JC Lucchesi PA Burghes AH Kaspar BK Early heart failure in the SMNDelta7 model of spinal muscular atrophy and correction by postnatal scAAV9-SMN delivery Hum Mol Genet 2010 5 3895 3905 10.1093/hmg/ddq300 20639395 PMC2947399

[B14] Dominguez E Marais T Chatauret N Benkhelifa-Ziyyat S Duque S Ravassard P Carcenac R Astord S Pereira de Moura A Voit T Barkats M Intravenous scAAV9 delivery of a codon-optimized SMN1 sequence rescues SMA mice Hum Mol Genet 2011 5 681 693 10.1093/hmg/ddq514 21118896

[B15] Oskoui M Kaufmann P Spinal muscular atrophy Neurotherapeutics 2008 5 499 506 10.1016/j.nurt.2008.08.007 19019300 PMC4514700

[B16] Brichta L Hofmann Y Hahnen E Siebzehnrubl FA Raschke H Blumcke I Eyupoglu IY Wirth B Valproic acid increases the SMN2 protein level: a well-known drug as a potential therapy for spinal muscular atrophy Hum Mol Genet 2003 5 2481 2489 10.1093/hmg/ddg256 12915451

[B17] Thurmond J Butchbach ME Palomo M Pease B Rao M Bedell L Keyvan M Pai G Mishra R Haraldsson M Andresson T Bragason G Thosteinsdottir M Bjornsson JM Coovert DD Burghes AH Gurney ME Singh J Synthesis and biological evaluation of novel 2,4-diaminoquinazoline derivatives as SMN2 promoter activators for the potential treatment of spinal muscular atrophy J Med Chem 2008 5 449 469 10.1021/jm061475p 18205293

[B18] Swoboda KJ Kissel JT Crawford TO Bromberg MB Acsadi G D’Anjou G Krosschell KJ Reyna SP Schroth MK Scott CB Simard LR Perspectives on clinical trials in spinal muscular atrophy J Child Neurol 2007 5 957 966 10.1177/0883073807305665 17761650 PMC3260051

[B19] Kobayashi DT Shi J Stephen L Ballard KL Dewey R Mapes J Chung B McCarthy K Swoboda KJ Crawford TO Li R Plasterer T Joyce C Chung WK Kaufmann P Darras BT Finkel RS Sproule DM Martens WB McDermott MP De Vivo DC Walker MG Chen KS SMA-MAP: a plasma protein panel for spinal muscular atrophy PLoS One 2013 5 e60113 10.1371/journal.pone.0060113 23565191 PMC3615018

[B20] Guest PC Gottschalk MG Bahn S Proteomics: improving biomarker translation to modern medicine? Genome Med 2013 5 17 10.1186/gm421 23445684 PMC3706758

[B21] Finkel RS Crawford TO Swoboda KJ Kaufmann P Juhasz P Li X Guo Y Li RH Trachtenberg F Forrest SJ Kobayashi DT Chen KS Joyce CL Plasterer T Candidate proteins, metabolites and transcripts in the Biomarkers for Spinal Muscular Atrophy (BforSMA) clinical study PLoS One 2012 5 e35462 10.1371/journal.pone.0035462 22558154 PMC3338723

[B22] El-Khodor BF Cirillo K Beltran JA Mushlin R Winberg ML Charney R Chomicova O Marino T Ramboz S Prediction of death in the SMNDelta7 mouse model of spinal muscular atrophy: insight into disease stage and progression J Neurosci Methods 2012 5 259 268 10.1016/j.jneumeth.2012.06.020 22750651

[B23] Crawford TO Paushkin SV Kobayashi DT Forrest SJ Joyce CL Finkel RS Kaufmann P Swoboda KJ Tiziano D Lomastro R Li RH Trachtenberg FL Plasterer T Chen KS Evaluation of SMN protein, transcript, and copy number in the biomarkers for spinal muscular atrophy (BforSMA) clinical study PLoS One 2012 5 e33572 10.1371/journal.pone.0033572 22558076 PMC3338744

[B24] Monani UR Sendtner M Coovert DD Parsons DW Andreassi C Le TT Jablonka S Schrank B Rossoll W Prior TW Morris GE Burghes AH The human centromeric survival motor neuron gene (SMN2) rescues embryonic lethality in Smn(-/-) mice and results in a mouse with spinal muscular atrophy Hum Mol Genet 2000 5 333 339 10.1093/hmg/9.3.333 10655541

[B25] Hsieh-Li HM Chang JG Jong YJ Wu MH Wang NM Tsai CH Li H A mouse model for spinal muscular atrophy Nat Genet 2000 5 66 70 10.1038/71709 10615130

[B26] Riessland M Ackermann B Forster A Jakubik M Hauke J Garbes L Fritzsche I Mende Y Blumcke I Hahnen E Wirth B SAHA ameliorates the SMA phenotype in two mouse models for spinal muscular atrophy Hum Mol Genet 2010 5 1492 1506 10.1093/hmg/ddq023 20097677

[B27] EuroBioBank http://www.eurobiobank.org/

[B28] Murray LM Gillingwater TH Parson SH Using mouse cranial muscles to investigate neuromuscular pathology in vivo Neuromuscular Disord 2010 5 740 743 10.1016/j.nmd.2010.06.013 20637618

[B29] Wishart TM Rooney TM Lamont DJ Wright AK Morton AJ Jackson M Freeman MR Gillingwater TH Combining comparative proteomics and molecular genetics uncovers regulators of synaptic and axonal stability and degeneration in vivo PLoS Genet 2012 5 e1002936 10.1371/journal.pgen.1002936 22952455 PMC3431337

[B30] Wishart TM Huang JP Murray LM Lamont DJ Mutsaers CA Ross J Geldsetzer P Ansorge O Talbot K Parson SH Gillingwater TH SMN deficiency disrupts brain development in a mouse model of severe spinal muscular atrophy Hum Mol Genet 2010 5 4216 4228 10.1093/hmg/ddq340 20705736 PMC2951867

[B31] ProteomeXchange http://proteomecentral.proteomexchange.org

[B32] Wishart TM Paterson JM Short DM Meredith S Robertson KA Sutherland C Cousin MA Dutia MB Gillingwater TH Differential proteomics analysis of synaptic proteins identifies potential cellular targets and protein mediators of synaptic neuroprotection conferred by the slow Wallerian degeneration (Wlds) gene Mol Cell Proteomics 2007 5 1318 1330 10.1074/mcp.M600457-MCP200 17470424 PMC2225590

[B33] Murray LM Lee S Baumer D Parson SH Talbot K Gillingwater TH Pre-symptomatic development of lower motor neuron connectivity in a mouse model of severe spinal muscular atrophy Hum Mol Genet 2010 5 420 433 10.1093/hmg/ddp506 19884170

[B34] BioGPS http://www.biogps.org

[B35] Fuller HR Man NT le Lam T Shamanin VA Androphy EJ Morris GE Valproate and bone loss: iTRAQ proteomics show that valproate reduces collagens and osteonectin in SMA cells J Proteome Res 2010 5 4228 4233 10.1021/pr1005263 20568814

[B36] Wu CY Whye D Glazewski L Choe L Kerr D Lee KH Mason RW Wang W Proteomic assessment of a cell model of spinal muscular atrophy BMC Neurosci 2011 5 25 10.1186/1471-2202-12-25 21385431 PMC3063191

[B37] Ni M Wei W Wang Y Zhang N Ding H Shen C Zheng F Serum levels of calreticulin in correlation with disease activity in patients with rheumatoid arthritis J Clin Immunol 2013 5 947 953 10.1007/s10875-013-9885-2 23532497

[B38] Gromov P Gromova I Bunkenborg J Cabezon T Moreira JM Timmermans-Wielenga V Roepstorff P Rank F Celis JE Up-regulated proteins in the fluid bathing the tumour cell microenvironment as potential serological markers for early detection of cancer of the breast Mol Oncol 2010 5 65 89 10.1016/j.molonc.2009.11.003 20005186 PMC5527961

[B39] Song MN Moon PG Lee JE Na M Kang W Chae YS Park JY Park H Baek MC Proteomic analysis of breast cancer tissues to identify biomarker candidates by gel-assisted digestion and label-free quantification methods using LC-MS/MS Arch Pharm Res 2012 5 1839 1847 10.1007/s12272-012-1018-6 23139137

[B40] Chen CN Chang CC Su TE Hsu WM Jeng YM Ho MC Hsieh FJ Lee PH Kuo ML Lee H Chang KJ Identification of calreticulin as a prognosis marker and angiogenic regulator in human gastric cancer Ann Surg Oncol 2009 5 524 533 10.1245/s10434-008-0243-1 19050968

[B41] Liu R Gong J Chen J Li Q Song C Zhang J Li Y Liu Z Dong Y Chen L Jin B Calreticulin as a potential diagnostic biomarker for lung cancer Cancer Immunol Immunother 2012 5 855 864 10.1007/s00262-011-1146-8 22083347 PMC11029700

[B42] Hsu WM Hsieh FJ Jeng YM Kuo ML Chen CN Lai DM Hsieh LJ Wang BT Tsao PN Lee H Lin MT Lai HS Chen WJ Calreticulin expression in neuroblastoma–a novel independent prognostic factor Ann Oncol 2005 5 314 321 10.1093/annonc/mdi062 15668290

[B43] Bernard-Marissal N Moumen A Sunyach C Pellegrino C Dudley K Henderson CE Raoul C Pettmann B Reduced calreticulin levels link endoplasmic reticulum stress and Fas-triggered cell death in motoneurons vulnerable to ALS J Neurosci 2012 5 4901 4912 10.1523/JNEUROSCI.5431-11.2012 22492046 PMC6620921

[B44] Bhattacharyya T Karnezis AN Murphy SP Hoang T Freeman BC Phillips B Morimoto RI Cloning and subcellular localization of human mitochondrial hsp70 J Biol Chem 1995 5 1705 1710 10.1074/jbc.270.4.1705 7829505

[B45] Ornatsky OI Connor MK Hood DA Expression of stress proteins and mitochondrial chaperonins in chronically stimulated skeletal muscle Biochem J 1995 5 119 123 7575442 10.1042/bj3110119 PMC1136127

[B46] Takahashi M Chesley A Freyssenet D Hood DA Contractile activity-induced adaptations in the mitochondrial protein import system Am J Physiol 1998 5 C1380 C1387 9612226 10.1152/ajpcell.1998.274.5.C1380

[B47] Deocaris CC Kaul SC Wadhwa R The versatile stress protein mortalin as a chaperone therapeutic agent Protein Pept Lett 2009 5 517 529 10.2174/092986609788167770 19442231

[B48] Hsu WM Lee H Juan HF Shih YY Wang BJ Pan CY Jeng YM Chang HH Lu MY Lin KH Lai HS Chen WJ Tsay YG Liao YF Hsieh FJ Identification of GRP75 as an independent favorable prognostic marker of neuroblastoma by a proteomics analysis Clin Cancer Res 2008 5 6237 6245 10.1158/1078-0432.CCR-07-4181 18829503

[B49] De Mena L Coto E Sanchez-Ferrero E Ribacoba R Guisasola LM Salvador C Blazquez M Alvarez V Mutational screening of the mortalin gene (HSPA9) in Parkinson’s disease J Neural Transm 2009 5 1289 1293 10.1007/s00702-009-0273-2 19657588

[B50] Osorio C Sullivan PM He DN Mace BE Ervin JF Strittmatter WJ Alzate O Mortalin is regulated by APOE in hippocampus of AD patients and by human APOE in TR mice Neurobiol Aging 2007 5 1853 1862 10.1016/j.neurobiolaging.2006.08.011 17050040

