# Supplementary material for: Label-free proteomics identifies Calreticulin and GRP75/Mortalin as peripherally accessible protein biomarkers for spinal muscular atrophy
Source: Genome Med. 2013 Oct 18;5(10):95. doi: 10.1186/gm498 (PMC3979019; doi:10.1186/gm498)
Supplement: Additional file 2: Table S2 — Peptide abundance (feature) output from Progenesis analysis software for proteins from P5 SMA versus control animals. [file gm498-S2.pdf]

Supplementary Table 2: Peptide abundance (feature) output from Progenesis analysis software for proteins from P5 SMA vs control animals

						Normalized abundance						Raw abundance										
#	m/z	Retention time (min)	Retention time window (min)	Mass	Charge	KO-R1	KO-R2	KO-R3	WT-R1	WT-R2	WT-R3	KO-R1	KO-R2	KO-R3	WT-R1	WT-R2	WT-R3	Score	Mass error (u)	Mass error (ppm)	Protein	Sequence
1814	529.61581	41.3863	0.684133333	1585.8256	3	74244.7099	74681.3527	72507.0063	101352.295	87431.2926	101106.298	84349.9163	80466.9929	80174.7943	101352.295	105722.169	100152.1	19.58	0.000301387	0.190050619	G6PI_MOUSE	VWFVSNIDGTHIAK
5602	889.501642	55.10476667	0.8905	1776.98873	2	72296.4852	74572.5471	80643.7897	65817.9735	53963.9262	69288.0567	82136.5251	80349.7581	89172.0619	65817.9735	65253.3337	68634.146	72.65	0.001431885	0.805793797	G6PI_MOUSE	TLASLSPETSLFIIASK
6273	663.932012	44.35215	0.522483333	3314.62368	5	54197.5683	52026.2781	54950.1961	70033.0396	67494.382	70827.414	61574.2233	56056.8067	60761.3097	70033.0396	81614.3995	70158.9756	9.29	0.001578538	0.476234535	G6PI_MOUSE	VDHQTGPIVWGEPGTNGQHAFYQLIHQGTK
10222	793.919912	41.43255	0.368183333	1585.82527	2	19403.5642	21421.1382	22861.7004	28780.1078	26294.7559	27543.3622	22044.5204	23080.6556	25279.3795	28780.1078	31795.6939	27283.4198	62.51	-2.94E-05	0.018542248	G6PI_MOUSE	VWFVSNIDGTHIAK
12364	559.320102	65.05146667	0.4668	1674.93848	3	30860.1377	25207.1865	27254.4259	24084.2559	21897.6273	17990.1812	35060.4109	27160.0128	30136.6461	24084.2559	26478.6734	17820.3977	45.07	0.00067735	0.40440305	G6PI_MOUSE	ILLANFLAQTEALMK
14576	612.809197	42.21608333	0.649966667	2447.20768	4	14009.6407	16244.0265	14613.7342	25135.4219	15881.349	20830.6639	15916.4474	17502.4677	16159.1713	25135.4219	19203.7725	20634.0731	3.54	1.004783156	410.7521727	G6PI_MOUSE	FNNFSLNLNTNHGHILVDYSK
15791	1028.48554	52.63701667	0.350733333	2054.95653	2	29307.8506	32033.6823	25493.2855	16110.9749	15800.4718	18100.1843	33296.847	34515.3642	28189.2609	16110.9749	19105.9757	17929.3626	89.61	0.002025129	0.985485994	GRP75_MOUSE	STNGDTFLGGEDFDQALLR
15823	784.889647	28.60001667	0.400516667	1567.76474	2	22585.6526	19929.1647	22408.46	12616.2861	10906.6017	14146.9399	25659.7125	21473.0974	24778.2079	12616.2861	13188.2939	14013.4273	39.29	0.001641693	1.047156299	GRP75_MOUSE	QAVTNPNNTFYATK
43217	671.314279	21.48748333	0.168866667	1340.61401	2	2720.94368	2944.48528	2881.08106	167.762047	0	12.2141246	3091.28249	3172.59754	3185.76222	167.762047	0	12.0988529	31.69	0.003105802	2.316706836	GRP75_MOUSE	ASNGDAWVEAHGK
5802	651.324303	30.10081667	0.489783333	1300.63405	2	56080.0815	61434.6171	53374.053	32931.3096	34279.1422	33996.7996	63712.9593	66194.0193	59018.4858	32931.3096	41450.4367	33675.9525	57.13	0.004152176	3.192434639	TBB5_MOUSE	ISVYYNEATGGK
8869	830.451471	51.2934	0.777733333	1658.88839	2	113896.051	97316.2129	100719.839	57491.4894	55763.4449	61651.8976	129398.073	104855.399	111371.201	57491.4894	67429.3168	61070.0537	40.89	0.00048903	0.294793918	TBB5_MOUSE	ALTVPELTQQVFDAK
2522	540.269041	25.92786667	1.138383333	1078.52353	2	22674.7639	83192.7892	87820.7883	102071.902	106618.237	112994.016	25760.9524	89637.8191	97108.0451	102071.902	128923.077	111927.628	2.58	1.011029405	938.2994677	PLEC1_MOUSE	SMVEEGTGLR
3368	509.780429	14.2701	0.652616667	1017.54631	2	100194.514	75718.2494	89244.284	5439.13679	7760.00722	7057.76022	113831.664	81584.2191	98682.0788	5439.13679	9383.42289	6991.15214	1.06	0.996105744	979.8883948	PLEC1_MOUSE	LLEAAAQSSK
3510	514.952686	45.86358333	0.60895	1541.83623	3	30619.0969	27071.3347	27767.1171	55197.4221	49630.4013	57598.0777	34786.5628	29168.5785	30703.5556	55197.4221	60013.223	57054.492	33.57	0.000827391	0.536627115	PLEC1_MOUSE	LGFHLPLEVAYQR
3731	395.200199	28.90508333	0.8894	1182.57877	3	13959.6897	14194.569	15295.544	29143.1931	26107.7486	30045.0395	15859.6978	15294.2367	16913.0841	29143.1931	31569.5642	29761.4874	8.34	2.017568281	1708.990843	PLEC1_MOUSE	QYDIDDAITK
5088	769.718951	54.6971	0.6637	2306.13502	3	45291.3313	42226.0777	42264.2389	79266.7461	71101.7079	82251.9075	51455.7873	45497.3748	46733.7824	79266.7461	85976.3882	81475.6497	35.44	0.001923478	0.834070467	PLEC1_MOUSE	GFFDPNTHENLTYLQLLER
5269	418.226682	34.66633333	0.44645	1251.65822	3	8195.66074	12316.1597	10736.812	16840.5266	15378.2152	16908.9339	9311.14552	13270.3051	11872.2553	16840.5266	18595.3818	16749.3547	9.17	0.001116604	0.892100456	PLEC1_MOUSE	SIQEELQHLR
6635	399.878739	13.26286667	0.4204	1196.61439	3	1777.03285	2115.87706	1757.58866	36720.4908	49229.0003	38775.9944	2018.89902	2279.7962	1943.45783	36720.4908	59527.8477	38410.0434	19.63	-0.000512455	0.42825389	PLEC1_MOUSE	LQHEATAATQK
7903	679.032433	67.7236	0.77295	2034.07547	3	53494.7678	44784.4786	53941.7362	72363.328	75682.6909	61190.5917	60775.7669	48253.9777	59646.2028	72363.328	91515.726	60613.1015	34.81	0.000868137	0.426796902	PLEC1_MOUSE	DGHNLISLLEVLSGDSLPR
8898	368.195977	19.78188333	0.594483333	1101.5661	3	13070.1189	13659.8718	14493.668	7665.96133	9143.937	8684.16973	14849.0504	14718.1159	16026.4077	7665.96133	11056.8747	8602.2123	4.61	1.008702551	916.5378844	PLEC1_MOUSE	QLAEGTAQQR
9757	684.879851	51.9869	0.455266667	1367.74515	2	15552.8897	16964.4825	16925.3304	17142.4632	14265.2885	15075.998	17669.743	18278.7382	18715.2242	17142.4632	17249.6276	14933.7173	67.98	0.000248201	0.181467112	PLEC1_MOUSE	IISLETYNLFR
11158	466.738132	16.57326667	0.608216667	931.461711	2	23093.1185	29460.2086	25958.5824	2711.10716	6695.45009	3917.7464	26236.2478	31742.5209	28703.7641	2711.10716	8096.15737	3880.77241	2.65	0.000710873	0.763180713	PLEC1_MOUSE	SLAQADAEK
11406	483.286071	38.24996667	0.4951	964.55759	2	12475.7311	13478.3582	12933.5876	12131.8001	11835.7	10511.6354	14173.7624	14522.5403	14301.3451	12131.8001	14311.7622	10412.4312	21.82	-0.001710475	1.773322619	PLEC1_MOUSE	LSYTQLLK
13130	515.782211	33.46035	0.4084	1029.54987	2	11261.5532	12937.076	11256.7219	7520.44033	8790.1725	6564.21574	12794.3266	13939.3244	12447.1469	7520.44033	10629.1017	6502.26552	36.54	0.004370045	4.24463472	PLEC1_MOUSE	VSLDEALQR
14719	723.409714	48.67913333	0.387016667	1444.80488	2	10399.3083	12051.5016	9070.09255	9617.64726	11001.7825	10892.2224	11814.7244	12985.1437	10029.2764	9617.64726	13303.3868	10789.4263	52.34	0.001076045	0.744769255	PLEC1_MOUSE	LQNVQIALDYLR
15380	773.903883	45.15666667	0.324633333	1545.79321	2	12570.2831	10706.343	10274.2581	13585.7103	10332.4753	11808.1835	14281.1835	11535.7743	11360.7852	13585.7103	12494.0587	11696.743	74.51	0.000613387	0.396810796	PLEC1_MOUSE	YSELTTLTSQYIK
16167	676.373004	38.20413333	0.447133333	2026.09718	3	9954.40964	7221.07767	10131.6972	17973.5042	18684.5879	16978.7478	11309.272	7780.5019	11203.1482	17973.5042	22593.457	16818.5097	43.79	0.001182145	0.583459391	PLEC1_MOUSE	LLEAQIATGGIIDPVHSHR
18749	986.197132	63.09681667	0.402583333	2955.56957	3	43753.6949	32789.6079	35447.9289	19262.2263	16908.367	18403.0321	49708.8682	35329.8521	39196.6315	19262.2263	20445.6457	18229.3522	55.55	0.00306715	1.037753609	PLEC1_MOUSE	DLLQPEVAVALLEAQAGTGHIIDPATSAR
20856	904.954731	46.9927	0.383216667	1807.89491	2	15496.1663	10919.8584	16722.4041	12760.5765	15337.1443	16017.7447	17605.2992	11765.831	18490.838	12760.5765	18545.7187	15866.5762	99.86	-0.000291162	0.161050372	PLEC1_MOUSE	VQSGSESVIQEYVDLR
21235	442.727207	10.78966667	0.570583333	883.439862	2	7415.17074	7454.30292	6331.40988	14980.7999	19815.5289	13806.718	8424.42555	8031.79534	7000.9715	14980.7999	23960.9941	13676.4161	10.6	-3.81E-05	0.043079972	PLEC1_MOUSE	THEEQLK
25034	800.943109	59.14395	0.358066667	1599.87167	2	19017.9212	12997.6881	13502.6981	4729.36461	6183.37964	5997.50411	21606.3888	14004.6322	14930.6405	4729.36461	7476.96032	5940.90226	4.77	-0.000234185	0.146377117	PLEC1_MOUSE	SDQLTGLSLLPLSEK
34004	592.968966	61.92461667	0.339983333	1775.88507	3	1938.06272	2760.95033	3669.84393	4006.05348	3692.32266	3939.57331	2201.84614	2974.84395	4057.93863	4006.05348	4464.76711	3902.39332	34.29	0.00086886	0.4892546	PLEC1_MOUSE	YLQDLLAWVEENQR
2491	586.337882	32.0909	0.4809	1170.66121	2	88021.0553	85638.1355	88683.0788	37368.9022	37319.3468	35644.6661	100001.315	92272.6088	98061.5248	37368.9022	45126.6608	35308.2672	69.68	0.000411036	0.351114895	THIL_MOUSE	LGTAAIQGAIEK
5431	837.956822	50.38	0.915683333	1673.89909	2	129127.699	120354.241	123680.267	56066.1128	52364.6108	59999.2615	146702.851	129678.207	136759.748	56066.1128	63319.4369	59433.0145	86.32	0.00029179	0.174317436	THIL_MOUSE	TPIGSFLGSLASQPATK
5479	703.400573	43.83568333	0.560366667	1404.78659	2	44630.5492	44218.6144	44264.3134	24436.8187	20535.4446	25985.0148	50705.0684	47644.2754	48945.3696	24436.8187	24831.5183	25739.7795	64.71	0.000192992	0.137381994	THIL_MOUSE	FASEITPITISVK
6113	558.97379	50.38	0.770666667	1673.89954	3	51848.2099	53583.4012	48040.4359	24140.0661	19888.0111	22714.2396	58905.1014	57734.5617	53120.826	24140.0661	24048.6398	22499.8725	22.73	0.00073948	0.441771032	THIL_MOUSE	TPIGSFLGSLASQPATK
6455	815.102349	61.03005	0.944766667	2442.28522	3	100915.378	98032.4909	80316.8033	36102.2175	33858.6528	39687.4738	114650.642	105627.168	88810.4959	36102.2175	40941.9797	39312.9206	57.39	0.001716719	0.702915346	THIL_MOUSE	IAAFADAAVDPIDFPLAPAYAVPK
8207	483.257522	30.94158333	0.57385	1929.00098	4	17456.7542	16512.0801	17097.9246	23910.8488	22654.4556	20938.4511	19832.7364	17791.2877	18906.0707	23910.8488	27393.8324	20740.8431	23.54	0.000680637	0.352844603	THIL_MOUSE	VNIHGGAVSLGHPIGMSGAR
23467	982.492447	61.6277	0.296983333	2944.45551	3	16470.5593	14248.5174	19386.8345	12817.8646	10428.2138	14956.3356	18712.3137	15352.3645	21437.0382	12817.8646	12609.8259	14815.1842	53.59	-0.000488705	0.165974665	THIL_MOUSE	ENGTITAANASTLNDGAAALVLMTAEAAQR
8403	502.774727	32.53166667	0.924816667	1003.5349	2	37501.979	40246.9985	36688.5111	25130.897	27372.5889	24605.2684	42606.2516	43364.9744	40568.4082	25130.897	33099.0127	24373.0546	7.35	0.001101025	1.097147434	CALR_MOUSE	LFPSGLDQK
11831	726.335984	41.34053333	0.45835	1450.65742	2	28445.6013	23107.8891	24245.6183	18947.4196	15450.5506	21447.9832	32317.2397	24898.0807	26809.65	18947.4196	18682.8499	21245.5665	49.53	0.009715103	6.697079176	CALR_MOUSE	EQFLDGDAWTNR
16323	496.738274	19.78188333	0.559583333	991.461995	2	28880.4622	16021.7839	19228.936	4452.79935	4799.54074	4085.73239	32811.2882	17263.0078	21262.4416	4452.79935	5803.61837	4047.17302	14.62	0.000895359	0.903070708	CALR_MOUSE	QIDNPDYK
2144	635.871864	39.95403333	0.601416667	1269.72918	2	121929.63	124169.536	121025.318	68838.4169	62588.1437	67458.4869	138525.076	133789.076	133824.032	68838.4169	75681.7622	66821.843	44.52	-2.40E-05	0.018933514	AATM_MOUSE	IAATILTSPDLR
2248	581.31993	45.33833333	0.852483333	2321.25061	4	133945.325	153373.707	134067.239	73576.3404	65268.9372	73473.2821	152176.188	165255.724	148245.168	73576.3404	78923.3852	72779.8731	28.76	0.001414954	0.609565633	AATM_MOUSE	ISVAGVTSGNVGYLAHAIHQVTK
2558	490.765724	30.1459	0.4916	979.516895	2	42060.6405	45425.3335	43375.9483	26612.5245	26233.5183	23756.1178	47785.3777	48944.4802	47963.0578	26612.5245	31721.6453	23531.9179	58.56	0.000794622	0.811239512	AATM_MOUSE	VGAFTVVCK
3296	725.407506	39.12165	0.69145	1448.80046	2	71500.346	70605.0449	76095.7486	58302.9443	54032.672	67445.633	81232.0259	76074.8894	84143.0546	58302.9443	65336.4613	66809.1104	74.18	0.001658487	1.144732318	AATM_MOUSE	FVTVQTISGTGALR
6368	779.407584	36.89286667	0.63915	1556.80061	2	72461.3237	70956.6084	72472.466	69952.8671	62724.796	67468.2551	82323.7992	76453.6889	80136.6013	69952.8671	75847.0025	66831.5189	71.69	-0.003985495	2.560048382	AATM_MOUSE	ASAELALGENNEVLK
10914	748.876892	50.38	0.435583333	1495.73923	2	27165.5157	25039.6191	23723.6161	22581.1224	19285.9894	27987.0419	30862.9258	26979.4638	26232.4448	22581.1224	23320.6734	27722.9123	51.07	0.001131455	0.756452885	AATM_MOUSE	EYLPIGGLAEFCK
17169	823.873579	52.97996667	0.442066667	1645.7326	2	16195.1706	15300.7744	19315.7999	14399.2755	11886.0599	17203.9517	18399.4427	16486.141	21358.4915	14399.2755	14372.6576	17041.5882	76.65	0.003204718	1.947293143	AATM_MOUSE	TCGFDFSGALEDISK
1320	714.396971	36.8017	0.593	1426.77939	2	224057.123	207025.538	202212.465	103853.26	94599.6951	100065.866	254552.811	223064.016	223596.913	103853.26	114390.221	99121.4875	81.33	0.001389428	0.973822477	THIM_MOUSE	VGVPTETGALTLNR
1824	603.31882	30.76858333	0.793983333	1806.93463	3	143775.578	121970.883	129031.79	113632.97	116896.176	108438.9	163344.406	131420.091	142677.208	113632.97	141351.189	107415.5	36.26	0.000930072	0.514723784	THIM_MOUSE	TNVSGGAIALGHPLGGSGSR
2100	575.965409	43.69521667	0.55795	1724.8744	3	88565.1502	82584.3095	80979.2851	97450.4025	82069.5331	97781.284	100619.465	88982.1998	89543.0366	97450.4025	99238.7138	96858.4666	28.91	0.000997633	0.578380447	THIM_MOUSE	LEDTLWAGLTDQHVK
2353	478.25886	21.91466667	0.83115	954.503167	2	107528.829	87395.573	85201.172	26149.3998	27717.5737	28205.2551	122164.228	94166.1968	94211.3981	26149.3998	33516.1693	27939.0662	18.05	-0.000333114	0.34899225	THIM_MOUSE	HNFTPLAR
3004	562.311169	42.9152	0.5517	1122.60778	2	55666.6316	55521.7566	54302.3382	45863.3245	40766.1284	45477.2756	63243.2361	59823.0835	60044.9393	45863.3245	49294.5189	45048.0808	29.02	0.000484617	0.431688991	THIM_MOUSE	TPFGAYGGLLK
3119	345.712976	30.94158333	0.927733333	689.411399	2	27520.2221	26677.2015	28226.7006	18892.529	17252.901	15925.7159	31265.9102	28743.9114	31211.7412	18892.529	20862.2572	15775.4159	13.91	0.000199149	0.288868908	THIM_MOUSE	LPSVFK
3420	402.752733	30.5928	0.703616667	803.490914	2	39816.9122	37401.5271	35604.9123	20201.6512	19053.559	17668.324	45236.2628	40299.0614	39370.2163	20201.6512	23039.618	17501.578	19.47	0.000413832	0.515042548	THIM_MOUSE	GVFIVAAK
5270	722.341909	50.03721667	0.731083333	1442.66926	2	72631.6785	65413.6583	65007.3889	29832.9161	31218.0973	36415.4913	82517.3404	70481.3208	71882.0746	29832.9161	37749.0124	36071.8177	81.79	0.001464099	1.014854979	THIM_MOUSE	DFSATDLTEFAAR
6981	497.258284	20.42445	0.253583333	992.502014	2	9015.62462	7640.50164	8685.07977	17998.1338	16586.1646	19073.8851	10242.712	8232.41906	9603.54757	17998.1338	20056.0376	18893.874	44.59	-0.001885722	1.899964246	THIM_MOUSE	YALQSQQR
14518	907.125629	58.09873333	0.524116667	2718.35506	3	45091.5161	37115.9443	47402.8104	23028.5553	21075.871	25490.2829	51228.776	39991.3542	52415.7701	23028.5553	25485.0034	25249.7167	59.76	0.00165801	0.609931623	THIM_MOUSE	VPPETIDSVIVGNVMQSSSDAAYLAR
15486	844.753451	49.01735	0.545016667	2531.23852	3	32859.8045	38459.9431	35396.9228	15070.4113	15079.9945	22231.057	37332.2458	41439.474	39140.2314	15070.4113	18234.7724	22021.2499	33.89	0.003424844	1.353032796	THIM_MOUSE	DGTVTAGNASGVSDGAGAVIIASEDAVK
41961	760.712519	63.62465	0.239733333	2279.11573	3	10007.8331	10295.543	11205.5499	4925.75649	3254.05984	1838.06563	11369.9668	11093.1492	12390.5634	4925.75649	3934.81845	1820.71876	13.34	0.001328111	0.582731264	THIM_MOUSE	DMDLIDVNEAFAPQFLSVQK
744	556.281337	32.93031667	0.70665	1110.54812	2	298972.446	297574.758	282097.833	131019.615	128881.542	122416.965	339664.614	320628.177	311930.348	131019.615	155843.929	121261.647	27.8	0.002320503	2.089515376	ANXA2_MOUSE	QDIAFAYQR
995	611.801962	26.62783333	0.8902	1221.58937	2	311071.882	284264.841	286888.171	106654.196	106623.124	106252.955	353410.866	306287.127	317227.275	106654.196	128928.986	105250.186	50.71	0.00167163	1.368407738	ANXA2_MOUSE	TPAQYDASELK
1074	771.928496	54.84931667	0.769433333	1541.84244	2	314376.124	292302.727	326381.468	200642.688	195116.583	212154.317	357164.837	314947.716	360897.083	200642.688	235935.53	210152.097	77.12	0.001138762	0.738572586	ANXA2_MOUSE	GVDEVTIVNILTNR
1161	825.993815	59.0406	1.063	1649.97308	2	336596.517	322961.94	344944.918	311285.561	288803.371	343110.574	382409.576	347982.129	381423.661	311285.561	349221.862	339872.445	90.3	0.001477564	0.895509045	ANXA2_MOUSE	SALSGHLETVILGLLK
1666	711.351784	39.17055	0.881866667	1420.68901	2	163829.652	158995.267	158736.536	114846.374	100918.182	121203.268	186127.974	171312.792	175523.302	114846.374	122030.554	120059.404	58.87	0.001614386	1.13634122	ANXA2_MOUSE	SLYYYIQQDTK
2603	518.267105	39.67976667	0.60215	1034.51966	2	59078.6095	58769.0994	53797.1209	55117.424	51477.7005	57173.1398	67119.6072	63322.0012	59486.2941	55117.424	62246.9825	56633.5645	8.85	-0.002242355	2.167528268	ANXA2_MOUSE	WISIMTER
3009	889.436433	56.05086667	0.724716667	1776.85831	2	144308.516	137203.521	148252.433	81989.1876	77867.159	100169.789	163949.88	147832.817	163930.479	81989.1876	94157.191	99224.4304	85.78	0.001912326	1.076241262	ANXA2_MOUSE	GLGTDEDSLIEIICSR
6495	436.226282	10.83126667	0.763816667	870.438011	2	71698.5762	74066.651	59942.2526	2765.65017	3262.60327	3066.86117	81457.2366	79804.6696	66281.2881	2765.65017	3945.1492	3037.91746	12.66	-0.000188579	0.216648409	ANXA2_MOUSE	SVCHLQK
7243	716.836808	50.0858	0.5866	1431.65906	2	28344.209	28788.7437	24561.3044	39052.9134	33551.4676	38362.4646	32202.0473	31019.0369	27158.7207	39052.9134	40570.5305	38000.4163	32.86	-3.66E-05	0.025558444	ANXA2_MOUSE	SYSPYDMLESIK
34797	988.499098	66.74435	0.443016667	1974.98364	2	9220.6133	7398.88368	7853.24927	22698.3935	25862.8728	28059.4771	10475.601	7972.08271	8683.74902	22698.3935	31273.4597	27794.6639	82.07	0.00034233	0.17333328	ANXA2_MOUSE	GDLENAFLNLVQCIQNK
5791	737.04376	46.2806	0.9371	2208.10945	3	44619.1323	51598.7316	49427.7359	58876.3989	53812.5848	58990.3799	50692.0975	55596.1377	54654.8362	58876.3989	65070.3312	58433.6542	59.08	0.00185182	0.838645529	PURA1_MOUSE	AHLVFDFHQAVDGLQEVQR
7571	743.909973	46.18921667	0.505583333	1485.80539	2	35272.272	39132.8799	40667.7856	22043.3286	19282.8475	18400.5672	40073.0664	42164.5439	44968.5004	22043.3286	23316.8742	18226.9106	83.21	0.001493544	1.005209224	PURA1_MOUSE	VVDLLATDADIVSR
16193	762.069785	62.19503333	1.42135	2283.18752	3	91913.2479	87271.6682	91596.9893	44831.1416	42261.511	40727.7192	104423.262	94032.6929	101283.588	44831.1416	51102.7398	40343.3486	67.86	0.001624662	0.711576795	PURA1_MOUSE	VGIGAFPTEQINEIGDLLQNR
21738	575.276892	47.71566667	0.485783333	1722.80885	3	6406.70908	8094.53136	7285.38943	6698.76427	6241.63646	6740.7781	7278.70545	8721.62293	8055.83665	6698.76427	7547.40464	6677.16155	13.6	0.001246087	0.723288345	PURA1_MOUSE	DGVYFMYEALHGPPK
24701	378.196797	8.104133333	0.59725	1131.56856	3	13336.9363	15262.2938	12048.8837	5075.06035	5274.01405	5672.59832	15152.1834	16444.6792	13323.0818	5075.06035	6377.35285	5619.06279	1.21	1.000561386	885.0077008	PURA1_MOUSE	LQQEAAATGSR
5414	654.355336	51.93663333	0.507566667	1306.69612	2	42905.518	43248.5316	38489.9628	36416.9025	28916.1396	28490.1443	48745.2487	46599.0393	42560.3677	36416.9025	34965.4785	28221.2666	45.59	0.000618167	0.473076846	S10AB_MOUSE	CIESLIAVFQK
6990	925.45621	64.3436	0.711133333	1848.89787	2	83620.0773	71206.6076	79831.5396	69789.7256	58167.3567	67012.8298	95001.3345	76723.0558	88273.9143	69789.7256	70336.1339	66380.3918	75.61	0.001066956	0.577076897	S10AB_MOUSE	TEFLSFMNTELAAFTK
688	656.82879	30.68021667	0.881933333	1311.64303	2	451237.724	400327.439	416063.043	227882.376	229927.07	239524.29	512654.225	431341.212	460062.697	227882.376	278028.47	237263.764	38.93	0.001126429	0.858793045	HBB2_MOUSE	VNPDEVGGEALGR
784	546.309039	33.91886667	0.528666667	1090.60353	2	148909.147	165425.476	154226.555	151380.098	143573.151	136659.259	169176.688	178241.156	170536.379	151380.098	173609.064	135369.528	41.36	0.001325322	1.215220694	HBB2_MOUSE	VITAFNEGLK
1078	511.260503	33.87345	0.851516667	1020.50645	2	97658.9368	111735.977	105932.078	98714.138	91893.9447	95816.3736	110950.978	120392.276	117134.647	98714.138	111118.42	94912.1002	34.67	0.000252617	0.247540942	HBB2_MOUSE	SAVSCLWAK
1240	414.259787	46.85031667	1.248966667	1653.01004	4	104947.071	106826.716	100193.181	144937.394	127643.405	137889.659	119231.077	115102.691	110788.848	144937.394	154346.77	136588.316	43.45	0.001042142	0.630451524	HBB2_MOUSE	LLGNAIVIVLGHHLGK
1900	552.010589	46.80476667	1.0972	1653.00994	3	117921.185	102118.355	109044.136	140018.074	130444.248	161875.95	133971.055	110029.568	120575.812	140018.074	157733.558	160348.235	5.23	0.000938024	0.567464346	HBB2_MOUSE	LLGNAIVIVLGHHLGK
7140	1003.96251	53.17895	0.541933333	2005.91047	2	80662.1681	69947.1708	88983.7174	50706.4943	49148.0566	56104.9464	91640.8339	75366.0492	98393.9567	50706.4943	59429.9704	55575.4522	117.96	0.001370512	0.683237533	HBB2_MOUSE	YFDSFGDLSSASAIMGNPK
3475	514.790577	35.47608333	0.673383333	1027.5666	2	40995.9315	41251.288	39768.7284	25083.5391	24718.6004	25114.3387	46575.7546	44447.0672	43974.3658	25083.5391	29889.8023	24877.3205	53.65	0.000400264	0.389526099	LMNA_MOUSE	LADALQELR
3743	584.959177	29.7021	0.799466667	1751.8557	3	81303.1736	82562.257	70411.3303	68004.4888	64545.0349	64819.5167	92369.0846	88958.4389	77857.4956	68004.4888	78048.0405	64207.7782	35.34	0.000701795	0.400601067	LMNA_MOUSE	NSNLVGAAHEELQQSR
4903	668.807656	27.46423333	0.613583333	1335.60076	2	53600.0579	51043.4271	42690.2095	23721.4302	27412.8878	29195.9921	60895.3877	54997.8132	47204.8004	23721.4302	33147.7422	28920.453	54.2	0.001759634	1.317486597	LMNA_MOUSE	AQNTWGCGSSLR
5819	746.378091	34.18276667	0.612366667	1490.74163	2	62129.534	53975.2733	51648.9218	30706.284	26368.5453	23915.0696	70585.7831	58156.7926	57110.9178	30706.284	31884.9203	23689.3695	67.71	0.001728594	1.159554296	LMNA_MOUSE	TALINSTGEEVAMR
6356	715.896357	41.25021667	0.455666667	1429.77816	2	40349.2422	38240.4184	38863.3206	25731.4226	25419.9743	28888.3325	45841.0465	41202.9425	42973.2089	25731.4226	30737.9055	28615.6969	82.04	0.000560324	0.391895874	LMNA_MOUSE	IDSLSAQLSQLQK
9770	850.442359	47.86013333	0.7757	2548.30525	3	77341.0435	71495.524	82204.9715	52736.3292	53152.8348	63920.637	87867.6818	77034.3549	90898.3423	52736.3292	64272.5597	63317.3818	54.26	-0.002153097	0.844912741	LMNA_MOUSE	AGQVVTIWASGAGATHSPPTDLVWK
14270	783.879761	33.46035	0.36505	1565.74497	2	19684.7189	17557.4778	15221.0863	10110.8633	10311.9556	10139.2849	22363.9421	18917.6734	16830.7522	10110.8633	12469.2461	10043.5947	69.68	0.001568552	1.001793643	LMNA_MOUSE	SVGGSGGGSFGDNLVTR
427	585.361313	48.19666667	1.065133333	1168.70807	2	286978.703	283599.148	307936.643	335209.816	299330.195	303819.211	326038.442	305569.862	340501.674	335209.816	361950.926	300951.897	53.21	0.001373455	1.175191973	VIME_MOUSE	ILLAELEQLK
959	547.268249	28.90508333	0.749483333	1092.52195	2	182998.611	198418.344	186912.057	120349.151	100324.105	116282.313	207905.958	213790.015	206678.45	120349.151	121312.195	115184.891	51.79	0.001945687	1.780916748	VIME_MOUSE	FADLSEAANR
1069	648.807671	32.1354	0.660683333	1295.60079	2	222376.405	217004.597	230185.182	140556.746	137721.022	137181.143	252643.335	233816.163	254527.811	140556.746	166532.653	135886.486	48.37	0.0014894	1.149584131	VIME_MOUSE	EEAESTLQSFR
1367	435.721784	27.1078	0.83635	869.429015	2	107972.08	106610.645	99822.769	58968.8621	62514.3873	58589.7754	122667.809	114869.879	110379.264	58968.8621	75592.5757	58036.8305	27.94	0.000714925	0.822293336	VIME_MOUSE	FANYIDK
1417	655.307193	36.75136667	0.9095	1308.59983	2	156452.334	139682.669	144473.598	155227.071	141358.905	148544.164	177746.553	150504.027	159752.024	155227.071	170931.592	147142.269	42.21	0.001233785	0.942829506	VIME_MOUSE	NLQEAEEWYK
1681	544.770596	21.40365	0.807716667	1087.52664	2	158872.655	206490.617	142451.087	72709.2369	78355.1172	68796.132	180496.297	222487.654	157515.627	72709.2369	94747.2314	68146.8639	46.49	0.000839416	0.771858579	VIME_MOUSE	QDVDNASLAR
1692	745.880642	38.24996667	0.680816667	1489.74673	2	141766.438	138685.562	128771.615	114584.444	102002.852	107751.542	161061.807	149429.673	142389.519	114584.444	123342.14	106734.63	68.47	0.002130125	1.429859501	VIME_MOUSE	QVQSLTCEVDALK
1992	779.454201	56.83246667	0.948233333	1556.89385	2	236408.539	181412.571	191683.402	194989.762	198133.31	204558.185	268585.338	195466.787	211954.377	194989.762	239583.364	202627.654	58.63	0.001249347	0.802461932	VIME_MOUSE	ISLPLPTFSSLNLR
3800	523.76902	26.89221667	0.670083333	1045.52349	2	38310.8189	35703.5633	38421.9517	22528.4957	26715.6167	22573.68	43525.1801	38469.5547	42485.1643	22528.4957	32304.6001	22360.6394	36.4	0.000886249	0.84766112	VIME_MOUSE	LQEEMLQR
5832	559.779867	26.62783333	0.347033333	1117.54518	2	26321.6034	24148.4461	27585.8938	16782.9951	20125.9531	16149.9942	29904.1513	26019.2507	30503.1675	16782.9951	24336.36	15997.5775	24.99	0.001481382	1.325569542	VIME_MOUSE	LQDEIQNMK
6737	841.934197	36.75136667	0.550166667	1681.85384	2	65595.2584	49024.4067	58897.349	43442.2974	43822.3964	47275.2901	74523.216	52822.3773	65125.8834	43442.2974	52990.1668	46829.1264	54.12	0.001540539	0.915977741	VIME_MOUSE	ETNLESLPLVDTHSK
24512	709.359561	60.46356667	0.539216667	2125.05685	3	16901.0397	14575.1692	13197.2797	6591.47238	8881.8553	9296.82086	19201.3853	15704.3223	14592.9234	6591.47238	10739.9648	9209.08149	35.22	-0.001047735	0.493038428	VIME_MOUSE	LLQDSVDFSLADAINTEFK
2058	516.277759	12.3121	0.703066667	1030.54096	2	9000.58228	6987.7239	7792.64346	211710.282	222020.663	187271.083	10225.6223	7529.06997	8616.73399	211710.282	268468.02	185503.7	5.73	0.995464962	966.897492	RRBP1_MOUSE	QSDELALVR
28490	991.193651	67.69873333	0.570716667	2970.55912	3	19875.9802	14488.1924	18097.5757	28838.1774	29636.4944	34883.1984	22581.2353	15610.6074	20011.4373	28838.1774	35836.5337	34553.9859	25.55	0.010023488	3.374287887	RRBP1_MOUSE	ETLLALLPGLSISAHQNYAEWLQEFK
29099	467.227039	11.50311667	0.710483333	932.439526	2	14652.8932	16646.7458	15718.8612	11865.4753	15581.7218	14140.7896	16647.251	17936.3861	17381.1681	11865.4753	18841.4625	14007.335	5.07	-0.005574332	5.978187559	RRBP1_MOUSE	ESEEALQK
216	593.838403	38.7979	1.013833333	1185.66225	2	745136.136	739217.541	723880.981	446229.07	403892.867	419738.62	846554.196	796485.475	800433.112	446229.07	488388.409	415777.309	38.41	0.001754009	1.47935166	TPM1_MOUSE	LVIIESDLER
443	566.308147	37.57168333	1.2723	1130.60174	2	352236.588	378504.841	344694.645	221722.214	201565.582	202308.132	400178.366	407827.996	381146.922	221722.214	243733.678	200398.836	42.25	0.001241436	1.098032772	TPM1_MOUSE	MEIQEIQLK
450	651.347755	39.95403333	1.111183333	1300.68096	2	554611.222	570062.607	564532.684	323812.729	293879.027	316455.022	630097.555	614225.937	624233.354	323812.729	355359.359	313468.457	33.34	-0.006442906	4.953462306	TPM1_MOUSE	QLEDELVSLQK
533	438.227954	15.38233333	1.01905	874.441354	2	575313.865	584809.554	527124.161	38680.8724	50967.8937	34840.4803	653617.967	630115.344	582868.792	38680.8724	61630.5226	34511.6709	33.37	0.001754165	2.00604513	TPM1_MOUSE	SLEAQAEK
605	769.865389	45.67366667	0.942266667	1537.71623	2	668012.564	621891.61	648828.729	300640.134	289588.41	306090.999	758933.585	670070.184	717443.907	300640.134	350171.133	303202.245	71.38	0.001425321	0.926908366	TPM1_MOUSE	SIDDLEDELYAQK
3186	879.915167	55.8946	0.57645	1757.81578	2	122675.493	128280.001	133422.876	77441.6455	74176.7742	88297.1255	139372.456	138217.983	147532.662	77441.6455	89694.7672	87463.8155	42.64	0.001680815	0.956196066	TPM1_MOUSE	AISEELDHALNDMTSI
1364	615.34848	54.34515	0.873383333	1228.68241	2	62717.5928	62580.2778	74747.7198	154231.602	131499.959	137051.55	71253.8807	67428.4355	82652.4685	154231.602	159010.127	135758.117	52.58	0.000907148	0.738310446	ACTN2_MOUSE	LASELLEWIR
2593	769.391019	41.25021667	0.594683333	1536.76749	2	88109.4473	87296.0579	95352.9087	125357.744	120103.748	132698.21	100101.738	94058.9721	105436.705	125357.744	145229.795	131445.862	49.26	0.000385084	0.250580647	ACTN2_MOUSE	FAIQDISVEETSAK
4120	711.356735	54.54461667	0.7672	1420.69892	2	32967.1684	29865.2486	30158.413	73354.878	67747.8728	68143.2462	37454.2226	32178.9397	33347.7367	73354.878	81920.9213	67500.1398	60.9	0.000317684	0.223611382	ACTN2_MOUSE	GYEEWLLNEIR
4287	686.378748	37.16205	0.723916667	1370.74294	2	36759.6268	37543.5315	34657.1422	47824.2399	41734.995	42973.5685	41762.8602	40452.067	38322.2172	47824.2399	50466.0751	42568.0026	52.38	0.002443888	1.782896375	ACTN2_MOUSE	QSILAIQNEVEK
4406	804.906598	43.74336667	0.465483333	1607.79864	2	56200.7791	63815.0637	59096.6704	67946.7956	56922.3438	67213.5667	63850.0846	68758.8815	65346.2836	67946.7956	68830.6607	66579.2342	82.24	0.000942818	0.586403185	ACTN2_MOUSE	CQLEINFNTLQTK
4867	877.416235	49.64921667	0.6721	1752.81792	2	71688.891	64958.0697	64583.3952	53918.9677	54715.5186	60735.4658	81446.2332	69990.4374	71413.2425	53918.9677	66162.1614	60162.2709	89.1	0.001316563	0.75111284	ACTN2_MOUSE	ETADTDTAEQVIASFR
4984	744.373677	33.96325	0.617566667	1486.7328	2	28774.9612	25025.0308	27295.5132	62086.0563	71403.977	50460.4714	32691.4278	26963.7453	30182.0785	62086.0563	86341.8929	49984.2473	53.61	0.001102006	0.741227403	ACTN2_MOUSE	NVNIQNFHTSWK
6279	761.368128	42.58828333	0.4681	1520.7217	2	38598.7364	35570.7645	37084.9799	63388.7845	52552.4081	57631.7123	43852.2851	38326.4677	41006.8045	63388.7845	63546.522	57087.8092	48.83	0.011003747	7.235923836	ACTN2_MOUSE	AGTQIENIEEDFR
6656	696.828445	31.02821667	0.441533333	1391.64234	2	18492.9182	19823.5984	14361.8459	42896.535	42613.6722	39911.1636	21009.9293	21359.3527	15880.6452	42896.535	51528.5741	39534.4994	41.63	0.001837673	1.32050815	ACTN2_MOUSE	TFTAWCNSHLR
6866	927.092516	58.71341667	0.687883333	2778.25572	3	72761.7312	61286.3382	63996.8692	71198.568	70404.0604	76240.9749	82665.0942	66034.253	70764.6899	71198.568	85132.7909	75521.4456	58.34	0.002719844	0.978976406	ACTN2_MOUSE	AIMTYVSCFYHAFAGAEQAETAANR
7861	900.428434	40.55545	0.507766667	1798.84231	2	46689.3047	39040.9894	41596.7105	44689.643	46921.2502	56389.9594	53044.0343	42065.5346	45995.6613	44689.643	56737.3098	55857.7754	104.26	0.001614246	0.897381242	ACTN2_MOUSE	ISSSNPYSTVTMDELR
8130	947.134283	54.44106667	0.666316667	2838.38102	3	56022.3077	57492.4164	51318.7429	90799.6604	74801.9773	77082.4103	63647.3221	61946.4123	56745.8217	90799.6604	90450.7646	76354.9399	76.49	0.002619715	0.922961785	ACTN2_MOUSE	VEQIAAIAQELNELDYHDAVNVNDR
12982	951.940665	53.82728333	0.508233333	1901.86678	2	33901.345	33749.3429	32147.2714	26967.6726	26435.2438	30224.2637	38515.5469	36363.9388	35546.9216	26967.6726	31965.5723	29939.0202	101.88	0.00167791	0.882244458	ACTN2_MOUSE	ACLISMGYDLGEAEFAR
19557	687.39806	61.86775	0.768083333	1372.78157	2	3044.04503	2455.91692	4797.02127	12969.742	9480.35059	11559.782	3458.36011	2646.1793	5304.31765	12969.742	11463.667	11450.6858	54.38	1.002067472	730.4872775	ACTN2_MOUSE	LMLLLEVISGER
39007	906.781333	72.09625	0.311066667	2717.32217	3	10716.4375	12369.0243	10039.4153	10851.1036	13050.1085	16918.2779	12175.017	13327.2652	11101.1073	10851.1036	15780.2285	16758.6105	37.05	0.002769081	1.019048639	ACTN2_MOUSE	IMTLVDPNGQGTVTFQSFIDFMTR
14949	752.442126	57.93126667	0.471283333	1502.8697	2	15049.7051	17202.1542	18192.5537	12234.4712	11202.017	13013.8927	17098.0716	18534.8226	20116.4594	12234.4712	13545.5109	12891.0732	87.57	-0.001100563	0.73230711	TCPB_MOUSE	LSSFIGAIAIGDLVK
33048	513.270352	14.07643333	0.358266667	1024.52615	2	2736.23312	2620.99266	2723.5304	3290.01423	5421.7806	4344.48953	3108.65293	2824.04363	3011.5502	3290.01423	6556.03259	4303.48812	14.57	0.007251429	7.077887044	TCPB_MOUSE	VAEIEHAEK
148	583.301821	37.79483333	1.4065	2329.17818	4	1194712.75	1354229.71	1222226.15	2107218.87	1849561.17	1832937.5	1357321.22	1459143.26	1351479.46	2107218.87	2236494.64	1815639.03	1.16	0.013276913	5.70028883	TCPB_MOUSE	VQDDEVGDGTTSVTVLAAELLR
13771	708.888409	49.6968	0.4825	1415.76227	2	11364.8793	10406.7997	10041.0843	8805.887	9751.64325	10930.4257	12911.7161	11213.0251	11102.9528	8805.887	11791.7149	10827.2691	61.08	0.000265343	0.187420608	POSTN_MOUSE	AAAITSDLLESLGR
17288	862.098161	68.67268333	0.474266667	2583.27265	3	45866.4751	46108.7577	44373.6006	59292.8481	50663.4787	58184.7035	52109.2121	49680.85	49066.2141	59292.8481	61262.4232	57635.5815	59.6	0.002252322	0.871887749	POSTN_MOUSE	VLTQIGTSIQDFLEAEDDLSSFR
17592	611.675771	54.23718333	0.766066667	1832.00548	3	6235.13663	6992.61328	7992.64401	17149.8766	13951.884	17019.3806	7083.78083	7534.33814	8837.88508	17149.8766	16870.658	16858.7591	13.31	0.001183357	0.645935462	POSTN_MOUSE	NGVIHLIDEVLIPDSAK
3250	408.260651	38.56855	0.599733333	814.50675	2	17811.721	18991.0886	18921.6314	19531.9302	17070.7737	19793.1934	20236.0166	20462.3475	20922.6388	19531.9302	20642.0283	19606.3939	25.19	0.000249937	0.306856531	HS90A_MOUSE	ALLFVPR
6889	604.819516	30.2369	0.755566667	1207.62448	2	30353.6596	30698.882	28022.4721	39349.9333	35359.6152	37639.9049	34484.9976	33077.1555	30985.915	39349.9333	42756.9477	37284.6758	17.53	0.00077808	0.644306933	HS90A_MOUSE	HIYFITGETK
13014	593.654363	50.62215	1.016783333	1777.94126	3	25094.9017	25005.0129	24200.9876	44892.8573	41878.8569	46613.5174	28510.4873	26942.1766	26760.2995	44892.8573	50640.0333	46173.5992	32.34	0.000959428	0.539628726	HS90A_MOUSE	HSQFIGYPITLFVEK
1553	757.39706	43.00466667	1.058983333	1512.77957	2	174883.947	169274.709	171091.611	139400.094	124817.093	133810.326	198686.833	182388.593	189184.954	139400.094	150929.186	132547.482	85.19	0.0011674	0.771692957	HS90B_MOUSE	GVVDSEDLPLNISR
1597	621.856462	41.11555	0.64315	1241.69837	2	128156.998	129160.069	137595.17	89823.1082	83299.2878	84045.4212	145600.03	139166.23	152146.185	89823.1082	100725.737	83252.237	56.05	0.000471303	0.379562971	HS90B_MOUSE	ADLINNLGTIAK
1833	638.325643	33.1474	0.577166667	1274.63673	2	104444.233	109069.638	89285.9256	87371.5727	74551.4216	82068.057	118659.798	117519.374	98728.124	87371.5727	90147.7919	81293.5343	62.8	0.001333687	1.046328046	HS90B_MOUSE	ELISNASDALDK
4747	576.283328	25.53516667	0.568933333	1150.5521	2	1697.42305	63654.0977	72375.3495	31761.7331	29612.9425	35394.5842	1928.45379	68585.4453	80029.2145	31761.7331	35808.0546	35060.5454	49.56	0.001503435	1.306708889	HS90B_MOUSE	YIDQEELNK
4955	764.376121	37.97948333	0.455133333	1526.73769	2	39012.4325	39997.4829	42344.5338	52221.4685	49838.1328	47996.6711	44322.288	43096.1286	46822.5688	52221.4685	60264.4125	47543.6993	54.25	0.001188225	0.77827781	HS90B_MOUSE	SLTNDWEDHLAVK
6954	726.320771	37.52498333	0.496133333	2175.94048	3	35394.953	35571.7502	34518.0768	45807.4287	45792.0291	42643.3075	40212.4451	38327.5298	38168.4453	45807.4287	55371.8523	42240.8584	50.75	0.002582287	1.186746516	HS90B_MOUSE	YHTSQSGDEMTSLSEYVSR
10107	571.284713	17.43098333	0.438766667	1140.55487	2	27504.6805	32534.7037	34936.4699	6407.1476	9406.15533	7095.25761	31248.2533	35055.2002	38631.0845	6407.1476	11373.9499	7028.29565	34.92	0.002572783	2.255734042	HS90B_MOUSE	LGIHEDSTNR
16545	816.721094	54.59641667	0.504166667	2447.14145	3	23768.9783	24001.2851	20662.2163	21458.1664	16584.8496	20766.2871	27004.0968	25860.6891	22847.2947	21458.1664	20054.4474	20570.3039	37.29	0.002553061	1.043284017	HS90B_MOUSE	GFEVVYMTEPIDEYCVQQLK
3971	497.592083	25.83858333	1.182466667	1489.75442	3	10285.6156	74583.2215	74241.1372	57535.1029	64336.0219	67721.6541	11685.5573	80361.2595	82092.3136	57535.1029	77795.3015	67082.5265	15.62	0.001918153	1.287564577	RTN4_MOUSE	YSNSALGHVNSTIK
4500	949.422584	38.15856667	0.639916667	4742.07654	5	142543.356	152676.373	138830.099	227370.379	200084.762	240315.09	161944.47	164504.366	153511.711	227370.379	241943.066	238047.1	1.12	-0.03196173	6.7399829	RTN4_MOUSE	SVPDHCELVDDSSPESEPVDLFSDDSIPEVPQTQEEAVMLMK
5202	605.789297	22.57396667	1.16465	1209.56404	2	86941.0819	71160.7864	76317.0765	38183.9569	41951.3409	45121.8411	98774.3503	76673.6849	84387.7884	38183.9569	50727.6812	44696.0007	1.34	0.00934046	7.722230497	RTN4_MOUSE	GESAMLVENTK
8460	365.715974	19.11476667	0.618316667	729.417396	2	1589.23891	2366.20608	1549.04077	16315.446	17310.4288	15483.1991	1805.54506	2549.51847	1712.8555	16315.446	20931.82	15337.0754	1.89	2.005796059	2757.443048	RTN4_MOUSE	TPELVK
18224	804.417194	30.1459	0.311583333	1606.81984	2	4166.5851	6299.96085	6170.65632	21912.9634	17864.3737	14472.932	4733.68547	6788.02521	6823.21787	21912.9634	21601.6517	14336.3428	63.93	-0.001764984	1.098431891	RTN4_MOUSE	HQAQIDHYLGLANK
2780	699.87492	39.81866667	0.882116667	1397.73529	2	83209.3696	87532.5899	88562.9219	149994.604	145158.795	139760.569	94534.7267	94313.8285	97928.661	149994.604	175526.429	138441.569	2.99	-0.004913733	3.515483465	NB5R3_MOUSE	STPAITLENPDIK
10923	699.876498	36.38491667	0.455983333	1397.73844	2	25713.9406	21071.4531	24148.2197	18981.1177	23042.4042	20668.8769	29213.7816	22703.88	26701.9512	18981.1177	27862.9409	20473.813	61.73	-0.001757672	1.257509459	NB5R3_MOUSE	STPAITLENPDIK
13105	640.361218	52.48773333	0.6963	2557.41577	4	17958.6327	19378.9648	18003.5685	28008.4373	23698.8048	26998.4778	20402.9239	20880.2729	19907.4886	28008.4373	28656.6625	26743.6779	0.55	-0.00113398	0.443408184	NB5R3_MOUSE	FALPSPQHILGLPIGQHIYLSTR
